# Mitochondrial DNA release contributes to neuropathic pain via a cGAS-STING-IRF3-CMPK2-associated immunometabolic feedback mechanism

**DOI:** 10.1186/s12967-026-08314-8

**Published:** 2026-05-22

**Authors:** Bo Wang, Hui Zeng, Hongrui Zhan, Yin Xu, Ziwei Hu, Jiahui Pang, Zhichao Zhang, Yan Ma, Wen Wu

**Affiliations:** 1 Center of Rehabilitation Medicine, Zhujiang Hospital, Southern Medical University, Guangzhou, 510282 China; 2 Department of Rehabilitation, Wuhan No. 1 Hospital, Wuhan, 430030 China; 3 Department of Rehabilitation, The Fifth Affiliated Hospital of Sun Yat-sen University, Zhuhai, 519000 China

**Keywords:** Neuropathic pain, Microglia, Neuroinflammation, Mitochondrial dysfunction, mtDNA, cGAS-STING, CMPK2, Immunometabolism

## Abstract

**Background:**

Innate immune-driven neuroinflammation in the spinal cord is a key mechanism underlying neuropathic pain (NP). Increasing evidence indicates that mitochondrial dysfunction and metabolic stress critically influence inflammatory responses. However, the mechanistic link between mitochondrial impairment and persistent neuroinflammation in NP remains incompletely understood.

**Methods:**

A peripheral nerve injury model was used to induce NP in mice. Mitochondrial integrity, mitochondrial DNA (mtDNA) release, and activation of the cGAS-STING-IRF3 pathway were examined in the spinal cord using immunofluorescence, molecular analyses, and single-cell RNA sequencing. Genetic silencing of CMPK2 was achieved by adeno-associated virus delivery, and pharmacological inhibition was performed using nordihydroguaiaretic acid (NDGA). Pain-related behaviors were assessed in vivo. Complementary in vitro experiments were conducted in BV2 cells and primary microglia to evaluate mitochondrial function and mtDNA-driven innate immune activation.

**Results:**

Peripheral nerve injury induced mitochondrial damage in the spinal cord, accompanied by cytosolic mtDNA release and activation of cGAS-STING-IRF3 signaling. IRF3 was observed to associate with the CMPK2 promoter and regulate CMPK2 transcription, consistent with a potential feedback mechanism that may exacerbate mitochondrial stress, enhance mtDNA release, and sustain innate immune activation. Single-cell RNA sequencing and immunofluorescence analyses revealed that CMPK2 was expressed in multiple spinal cord cell types, with microglia representing a major population contributing to CMPK2 upregulation in the spinal dorsal horn after nerve injury. Genetic silencing or pharmacological inhibition of CMPK2 was associated with reduced cGAS-STING signaling, improved mitochondrial homeostasis, decreased microglial activation, and attenuation of NP-like behaviors in vivo. Consistently, CMPK2 knockdown in microglia attenuated mtDNA-induced innate immune activation and improved mitochondrial function in vitro.

**Conclusions:**

These findings support a model in which an mtDNA-cGAS-STING-IRF3-CMPK2-associated immunometabolic feedback mechanism operates within the spinal cord microenvironment, with notable microglial involvement, linking mitochondrial dysfunction to sustained neuroinflammation and NP. Targeting mitochondrial immunometabolism may represent a potential therapeutic strategy for chronic inflammatory conditions characterized by persistent innate immune activation.

**Supplementary Information:**

The online version contains supplementary material available at 10.1186/s12967-026-08314-8.

## Introduction

Neuropathic pain (NP) is a chronic neurological disorder resulting from lesions or diseases of the somatosensory system and is clinically characterized by spontaneous pain, hyperalgesia, allodynia, and other sensory abnormalities [1, 2]. Owing to its high prevalence and poor responsiveness to conventional analgesics, NP remains a major clinical challenge. Epidemiological studies estimate a global NP prevalence of approximately 7%-10% in 2020, while a recent survey conducted in the United States in 2022 reported a substantially higher prevalence of 14.6%, highlighting considerable variability across populations [3]. Similar to other forms of chronic pain, NP markedly diminishes patients’ quality of life and imposes a substantial socioeconomic and psychological burden [4, 5]. Despite its clinical significance, current therapeutic options provide limited and often inadequate relief, underscoring the need to elucidate the molecular mechanisms that drive NP pathogenesis.

Growing evidence indicates that neuroinflammation plays a central role in the initiation and maintenance of NP [6, 7]. Microglia, the resident macrophage-like immune cells of the central nervous system, are widely recognized as key regulators of neuroinflammatory responses [8]. Following nerve injury, microglia in the spinal dorsal horn (SDH) undergo proliferation and activation, thereby exacerbating neuroinflammation and mechanical hypersensitivity through the release of pro-inflammatory mediators such as interleukin-1β (IL-1β), interleukin-6 (IL-6), and tumor necrosis factor-α (TNF-α) [9]. Consistent with these observations, our previous studies and those of others have demonstrated that suppressing microglia-mediated neuroinflammation effectively alleviates NP [10–12].

Mitochondria are the primary site of oxidative phosphorylation and ATP production and play a central role in maintaining cellular homeostasis. Under conditions of environmental stress or pathogenic stimulation, mitochondrial destabilization results in the release of reactive oxygen species (ROS), mitochondrial DNA (mtDNA), and other cytotoxic factors into the cytosol [13]. Accumulating evidence indicates that cytosolic mtDNA derived from damaged mitochondria acts as a potent trigger of inflammatory signaling [14, 15]. As a mitochondrial danger-associated molecular pattern (mtDAMP), mtDNA engages pattern-recognition receptors and initiates innate immune responses [16]. In the context of the central nervous system, inhibition of microglial mtDNA release has been shown to attenuate neuroinflammation and improve functional outcomes in experimental models of spinal cord injury [17]. Moreover, the cyclic GMP-AMP synthase (cGAS)-Stimulator of interferon genes (STING) pathway has been identified as a major sensor of cytosolic mtDNA [18]. Upon binding mtDNA, cGAS catalyzes the production of cyclic GMP-AMP, leading to STING activation and subsequent recruitment of TANK-binding kinase 1 (TBK1). Activated TBK1 phosphorylates interferon regulatory factor 3 (IRF3) and promotes nuclear factor-κB signaling, thereby driving the expression of type I interferons and pro-inflammatory cytokines [19]. Although increasing evidence implicates the cGAS-STING pathway in multiple pain-related conditions [20–22], the upstream mechanisms that regulate mtDNA-cGAS-STING signaling in NP remain poorly defined.

Cytosine/uridine monophosphate kinase 2 (CMPK2), a member of the mitochondrial novel nucleoside monophosphate (NMP) kinase family, catalyzes the sequential phosphorylation of deoxycytidine monophosphate (dCMP) to deoxycytidine diphosphate (dCDP) and subsequently to deoxycytidine triphosphate (dCTP), thereby serving as a rate-limiting enzyme for mitochondrial DNA synthesis and the generation of oxidized mtDNA fragments [23]. Beyond its metabolic role, CMPK2 has emerged as a critical regulator of innate immune responses [24, 25]. Both bacterial pathogen-associated molecular patterns (PAMPs), such as lipopolysaccharide (LPS), and viral PAMPs, including double-stranded DNA (dsDNA) and double-stranded RNA (dsRNA), robustly induce CMPK2 expression. Consistent with its immunoregulatory function, CMPK2 has been implicated in the pathogenesis of multiple inflammation-associated disorders. Pharmacological inhibition or genetic ablation of CMPK2 suppresses pathogenic inflammatory cascades and ameliorates disease severity in models of sepsis, pneumonia, and viral infection [26–28]. Notably, CMPK2 has also been linked to the regulation of cGAS-STING signaling [29–31]. LPS-stimulated macrophages exhibit marked upregulation of CMPK2, whereas deletion of IRF3 significantly attenuates its expression [32]. In addition, IRF1 has been identified as a direct transcriptional activator of *Cmpk2* transcription [16]. Collectively, these findings position CMPK2 at the intersection of mitochondrial metabolism and innate immune signaling, suggesting its potential involvement in mtDNA-driven neuroinflammatory responses.

Despite recent advances, the mechanisms by which CMPK2 regulates neuroinflammatory processes in NP remain incompletely understood. In this study, we hypothesized that an IRF3-CMPK2-STING-associated immunometabolic feedback mechanism contributes to mtDNA release and persistent neuroinflammation during NP. To address this, we integrated bioinformatic analyses of spinal cord tissues from NP mouse models with transcriptomic profiling of LPS-stimulated microglia to identify differentially expressed genes associated with mitochondrial and innate immune pathways. Using a spared nerve injury (SNI) model, we characterized the temporal dynamics of CMPK2 expression, cytosolic mtDNA accumulation, and activation of the cGAS-STING-IRF3 axis. Single-cell RNA sequencing and immunofluorescence analyses were employed to define the cellular distribution of CMPK2 in the injured spinal dorsal horn. Functional relevance was evaluated through adeno-associated virus (AAV)-mediated knockdown of *Cmpk2*, followed by assessment of mitochondrial integrity, mtDNA release, inflammatory signaling, and pain-related behaviors in vivo. Complementary mechanistic studies were conducted in LPS-stimulated microglia, including chromatin immunoprecipitation and dual-luciferase reporter assays to examine IRF3-associated regulation of *Cmpk2* expression. Finally, molecular docking and molecular dynamics simulations were performed to evaluate the interaction between CMPK2 and NDGA, and the in vivo effects of NDGA were further examined in SNI mice.

## Materials and methods

### Analysis of RNA-seq and scRNA-seq

RNA-seq and scRNA-seq data were sourced from the NCBI Gene Expression Omnibus (GEO) database. Analyses were performed using DESeq2 or limma R packages, with differentially expressed genes (DEGs) defined by an adjusted p-value < 0.05 and absolute log2 (fold change, FC) ≥ 1.0. scRNA-seq data were normalized and clustered via Seurat (v5.0.1). The top 2,000 highly variable genes were selected for principal component analysis (PCA), and optimal dimensionality was determined using the JackStraw function in Seurat. The first ten principal components were utilized for clustering. Graph-based algorithms and Uniform Manifold Approximation and Projection (UMAP) were employed for cell clustering and visualization in PCA-reduced space. Cell clusters were annotated based on canonical marker genes, and mean *Cmpk2* expression per cell was quantified.

### Animals

Male C57BL/6 mice (6–8 weeks, 20–30 g) were obtained from the Experimental Animal Center of Zhujiang Hospital, Southern Medical University. All animals were housed under controlled conditions with environmental parameters: temperature maintained at 22 ± 2 °C, humidity at 55 ± 10%, and a 12-hour light/dark cycle. Mice were provided with standard laboratory chow and filtered water ad libitum.

### NP model

The SNI surgical modeling procedure was performed as described in our laboratory’s previously published work [11]. Briefly, following anesthesia, exposure of the left sciatic nerve was performed, with selective ligation of the tibial and common peroneal nerve branches while sparing the sural nerve. For the sham-operated mice, the sciatic nerve and its trifurcation were exposed without further manipulation. Time points for molecular analyses were selected based on the temporal profile of microglial activation and innate immune signaling following SNI, whereas behavioral assessments were extended to later stages to capture the establishment and persistence of NP.

### Measurements of mechanical pain thresholds

As described in our previously published work, the von Frey test was employed to quantify mechanical pain thresholds in mice [11]. In brief, each mouse was acclimated for 30 min in a transparent behavioral chamber prior to testing and subsequently positioned on a perforated metal grid floor. Subsequently, von Frey filaments were applied perpendicularly to the plantar surface of the hind paw with sustained pressure for 3–4 s, maintaining a minimum 10-minute inter-stimulus interval between consecutive tests. A rapid withdrawal of the hind paw upon filament application was defined as a positive response. The final mechanical sensitivity was quantified by converting the response pattern to a 50% paw withdrawal threshold (PWT) using Dixon’s up-down method [33].

### AAV viral vectors construction and delivery in mice

AAV vectors encoding pAAV-U6-shRNA-*Cmpk2* (to induce RNA interference-mediated knockdown) were intrathecally injected into mice, achieving specific silencing of *Cmpk2*. The effective shRNA target sequences were as follows: Mouse *Cmpk2* (GenBank NM_020557.4): 5′-GGAAGAGTGCACATCCTTTAT-3′. Scrambled shRNA sequences were engineered into non-targeting control AAV vectors. The intrathecal injection protocol was performed as follows: following anesthesia induction, a midline skin incision was made to expose the L5-L6 spinous processes, after which the viral solution was delivered into the intrathecal space using a microinjection syringe. All AAV preparations in this study were quantified at titers of 1–2 × 10^^12^ vg/mL, aliquoted, and cryopreserved at − 80 °C until further use.

### Assessment of NDGA in vivo

To investigate the improvement effect of NDGA on mice with NP models, a total of 36 mice were randomly divided into 3 groups: Sham plus vehicle group, SNI plus vehicle group, SNI plus NDGA (2 µg, i.t.) group.

### BV2 and primary microglia cell cultivation

The BV2 microglial cell line was cultured in Dulbecco’s modified Eagle’s medium (DMEM, VivaCell, China) supplemented with 10% fetal bovine serum under standard culture conditions (37 °C, 5% CO₂, 95% relative humidity). The culture medium was changed every 2–3 days. When the cells reach 80% confluence, sub-culturing was carried out.

Primary microglia were isolated from the cerebral cortices of 1-2-day-old C57BL/6 neonatal mice. Briefly, meninges were removed, and cortical tissues were mechanically dissociated and enzymatically digested. The resulting cell suspension was filtered through a 70 μm cell strainer and centrifuged. Cells were resuspended in DMEM/F12 (Gibco, USA) supplemented with 10% FBS and 1% penicillin-streptomycin and seeded into T25 culture flasks. Mixed glial cultures were maintained for 10–14 days, after which microglia were harvested by shaking at 200 rpm for 6 h at 37 °C to obtain a highly enriched population for subsequent experiments.

### Drug administration

LPS (Cat. L2630, Sigma-Aldrich) was added to the culture medium to establish an LPS-induced BV2 microglial activation model. To inhibit the cGAS–STING signaling pathway, BV2 microglia were treated with C-176 (1 µM; Cat. S6575, Selleck). To activate IRF3 signaling, BV2 microglia were treated with KIN1148 (1 µM; Cat. S0289, Selleck).

### mtDNA isolation and transfection

According to the manufacturer’s protocol of the mitochondrial DNA isolation kit (K280-50, BioVision), mtDNA was isolated from BV2 microglia. The purified mtDNA was resuspended in Tris-EDTA (TE) buffer (10 mM Tris-HCl, 1 mM EDTA, pH 8.0) and stored at -20 °C until use. BV2 microglia were seeded in 6-well plates, and mtDNA (1 µg per well) was transfected using Lipofectamine 3000 transfection reagent (TL301-01, Vazyme, China) according to the manufacturer’s instructions.

### Detection of cytosolic mitochondrial DNA release

SDH tissue: after experimental treatments, SDH tissue (5 µg) was homogenized on ice in 200 µL digitonin lysis buffer (50 mM HEPES pH 7.4, 150 mM NaCl, 25 µg/mL digitonin; Sigma-Aldrich). Homogenates were incubated for 10 min on a roller mixer at 4 °C, followed by sequential centrifugation: twice at 980 × g for 5 min to pellet nuclei and debris, and the combined supernatant centrifuged at 17,000 × g for 25 min to remove mitochondria. Cytosolic DNA was extracted from the final supernatant using the FastPure Cell/Tissue DNA Isolation Kit (Nanjing Vazyme Biotech, China). Total DNA was isolated from separate tissue aliquots using the same kit.

BV2 microglial cells: stimulated BV2 cells were harvested and divided equally. Total DNA was extracted from one aliquot (FastPure Cell/Tissue DNA Isolation Kit). The second aliquot was gently resuspended in digitonin lysis buffer (50 mM HEPES pH 7.4, 150 mM NaCl, 25 µg/mL digitonin), incubated for 10 min at 4 °C on a rotator, and centrifuged at 980 × g for 3 min to pellet intact cells/nuclei. The supernatant was further cleared at 17,000 × g for 10 min, and cytosolic DNA extracted from the resulting supernatant (FastPure Cell/Tissue DNA Isolation Kit).

Cytosolic and total DNA extracts were analyzed by qPCR using SYBR Green Pro Taq HS Premixed qPCR Kit (AG11701, Accurate Biology, China) with primers specific for mitochondrial DNA (Dloop1-3) and nuclear DNA (Tert). The mitochondrial D-loop region, a non-coding control region uniquely encoded by the mitochondrial genome and present in multiple copies, was used as a surrogate marker for mtDNA abundance. Primer sequences (Tsingke Biotechnology) are listed in Supplementary Table 1.

### Western blot analysis

Total protein was extracted from SDH tissue or BV-2 microglial cells using RIPA lysis buffer supplemented with protease/phosphatase inhibitors (Beyotime Biotechnology, China). Protein concentrations were determined via BCA assay (Beyotime Biotechnology, China). Proteins were separated by SDS-PAGE and transferred to PVDF membranes (Millipore, USA). After blocking with 5% non-fat milk in TBST, membranes were incubated overnight at 4 °C with the primary antibodies. Membranes were then incubated with HRP-conjugated secondary antibodies for 1 h at room temperature. Protein bands were visualized using chemiluminescence detection system (Fdbio Science, China) and quantified with ImageJ software (version 1.53a, National Institutes of Health, USA). GAPDH served as the internal reference for normalization. The above antibodies utilized in this study are presented in Supplementary Table 2.

### Quantitative real-time PCR (qRT-PCR)

Total RNA was extracted from SDH tissue or BV-2 microglial cells using the Steady Pure Universal RNA Extraction Kit (AG21017, Accurate Biology, China). cDNA was synthesized from 1 µg RNA with a cDNA synthesis kit (AG11705, Accurate Biology, China). qPCR was performed by using SYBR Green Pro Taq HS Premixed qPCR Kit (AG11701, Accurate Biology, China). Reactions included primers for target genes *(Ccl5*, *Cfb*, *Gbp2*, *Il1b*, *Rsad2*, *Cmpk2*, *Ifit1*, *IL-1β*, *TNF-α*, *IL-6*) and the endogenous control GAPDH (primer sequences in Supplementary Table 1). Gene expression was quantified by the 2^−ΔΔCt^ method, normalized to GAPDH.

### mtROS measurement

MitoSOX Red dye (Invitrogen; M36008) was employed to measure mitochondrial reactive oxygen species (mtROS) following the manufacturer’s protocol. BV2 microglial cells were first treated with LPS at a concentration of 1 µg/ml for 24 h. After treatment, cells were stained with 5 µM MitoSOX Red dye for 10 min, then washed with PBS and fixed in paraformaldehyde for another 10 min. Subsequently, the cells were counterstained with DAPI for 10 min to label nuclei. Finally, the stained cells were visualized and photographed using a Nikon confocal microscope (AXNIS-Elements 5.4, Japan).

### Mitochondrial membrane potential (MMP) assay

Mitochondrial membrane potential (MMP) was assessed using a JC-1 staining kit (Beyotime, China). For cellular experiments, BV2 or primary microglia were incubated with JC-1 working solution in serum-free medium at 37 °C for 30 min. After washing, fluorescence signals were visualized using a confocal microscope (AXNIS-Elements 5.4, Japan), and the red/green fluorescence ratio was used to evaluate changes in mitochondrial membrane potential.

### Immunofluorescence

For the in vivo study, mice were transcardially perfused with 4% paraformaldehyde (PFA). L4-L6 spinal cord segments were post-fixed in 4% PFA at 4 °C overnight, followed by dehydration in 30% sucrose solution at 4 °C for 72 h. Tissues were then sectioned at a thickness of 15 μm using a cryostat (Leica, Germany). Sections were permeabilized with 0.3% Triton X-100 for 15 min and blocked with 5% goat serum for 1 h at room temperature. Subsequently, sections were incubated with primary antibodies overnight at 4 °C and then with the corresponding secondary antibodies for 2 h at room temperature. Images were acquired using a Nikon confocal microscope (AXNIS-Elements 5.4, Japan) and analyzed with ImageJ software (version 1.53a, National Institutes of Health, USA). For quantitative analysis, CMPK2-positive cells in the spinal dorsal horn were quantified within a 500 μm × 500 μm measuring frame. To avoid double counting, every third transverse Sect.  (40 μm apart) was analyzed. CMPK2-positive cells were identified based on discrete, morphologically defined CMPK2-immunoreactive cell body-like profiles, and results were expressed as the number of positive cells per square millimeter.

For the in vitro study, BV-2 microglia (2 × 10⁵ cells/well) grown on coverslips were incubated with MitoTracker (200 nM) at 37 °C for 30 min (Invitrogen). Cells were fixed with 4% PFA for 15 min at room temperature, permeabilized with 0.1% Triton X-100 for 10 min, and blocked with 5% goat serum. Samples were then incubated with primary antibodies, including anti-dsDNA antibody, overnight at 4 °C. Nuclei were counterstained with Hoechst to distinguish nuclear from extranuclear signals. After washing with PBS, coverslips were incubated with Alexa Fluor 488-conjugated secondary antibodies for 1 h at room temperature. Confocal images were obtained using a Nikon confocal microscope (AXNIS-Elements 5.4, Japan), and extranuclear dsDNA-mitochondrial co-localization was quantified using ImageJ software (version 1.53a, National Institutes of Health, USA).

### siRNA construction and transfection

siRNAs targeting mouse *Cmpk2* were purchased from Obio Technology (Shanghai, China). BV2 microglia were transfected with 20 nM siRNA and Lipo3000 Transfection Reagent (TL301-01, Vazyme, China) according to the manufacturer’s protocol. After 48 h, qRT-PCR was used to evaluate the knockdown efficiency of *Cmpk2*. Scramble siRNA was used as a negative control (siNC). The siRNA sequences used in this study are shown in Supplementary Table 1.

### Chromatin immunoprecipitation (ChIP) assay

Chromatin immunoprecipitation was performed to assess IRF3 binding to the *Cmpk2* promoter by using SimpleChIP^®^ Enzymatic Chromatin IP Kit (Cell signaling Tecnology, 9003). BV-2 microglia were crosslinked with 1% formaldehyde, lysed, and sonicated to generate 200–500 bp chromatin fragments. Lysates were immunoprecipitated overnight at 4 °C with 1 µg of anti-IRF3 antibody (Cell Signaling Technology, MA, USA). Normal rabbit IgG served as a negative control. Immune complexes were captured using protein A/G agarose beads, washed sequentially with low-salt, high-salt, and LiCl buffers, then eluted and reverse-crosslinked. Purified DNA was analyzed by quantitative PCR (qPCR) using primers flanking the predicted IRF3-binding site in the *Cmpk2* promoter region.

### Dual-luciferase reporter assay

BV-2 microglial cells (1 × 10⁵ cells/well) were co-transfected with 375 ng firefly luciferase vector and 125 ng Renilla control vector. After 48 h transfection, cells were stimulated with LPS (1 µg/mL, 24 h) to activate endogenous IRF3. Luciferase activities were measured using the Dual-Luciferase^®^ Assay System (Promega). Data expressed as relative luminescence (Firefly/Renilla) normalized to unstimulated WT controls.

### Molecular docking

The NDGA compound was obtained from PubChem (https://pubchem.ncbi.nlm.nih.gov/compound/4534). And the CMPK2 protein was obtained from the AlphaFold database (https://alphafold.ebi.ac.uk/entry/Q3U5Q7). Molecular docking was performed using AutoDock Vina 1.2.3, and AutoDockTools 1.5.7 was employed to complete the pre-processing, operation, and analysis of the docking results. Kollman charges, solvation parameters, and polar hydrogen atoms were added to the protein structure, while Gasteiger charges were assigned to the ligands. The Lamarckian genetic algorithm was used for conformational search, and 100 docking operations were carried out for each ligand to obtain the optimal conformation. The conformation with the best binding was selected to create a 3D image using PYMOL 2.6.0 and a 2D binding diagram using Discovery Studio 2019.

### Molecular dynamics simulation

The complex of structures of NDGA-CMPK2 were prepared by GROMACS 2024-2, ORCA, Multiwfn and sobtop. We utilize GROMACS as the backend engine with a common simulation setting for all complexes, providing a capability of high-throughput MD simulations. Specifically, this pipeline comprises four stages: preparation, minimization, equilibration, and production simulations. In the system preparation stage, a protein topology is generated with pdb2gmx with the amber14sb force field with the tip3p explicit water model; the ligand parameter and topology are generated with sobtop. Then, a cubic simulation box is used with a minimum distance of 1.2 nm between the protein-ligand complex and the box boundaries. Once the simulation box is prepared, an energy minimization process is carried out to remove the atomic clashes and optimize the geometry of all molecules. In the equilibration simulation stage, a thermostat is applied to heat the system from 0 to 300 K within 100 ps. The heated system is then further equilibrated to 1 bar in an NPT ensemble for another 100 ps. During the equilibration stage, the bonds for molecules are constrained. For the final production, the leap-frog algorithm is used for integrating Newton’s equations of motion and a Particle Mesh Ewald (PME) method is used for calculating long-range electrostatic interactions. The LINCS algorithm is adopted for resetting all bonds to their correct lengths after an unconstrained update. After relaxation, the systems were submitted to 100 ns production simulations with an integration time step of 2 fs.

### CMPK2 enzymatic activity assay

CMPK2 kinase activity was assessed in the presence of increasing concentrations of NDGA using a commercially available kinase assay kit according to the manufacturer’s instructions. The IC₅₀ value was calculated by nonlinear regression analysis.

### Cellular thermal shift assay (CETSA)

BV2 cells were treated with NDGA or DMSO control and subjected to heat treatment at the indicated temperatures. Soluble protein fractions were collected after centrifugation and analyzed by Western blot using anti-CMPK2 antibody.

### Statistical analysis

Statistical analysis in bioinformatics was performed using R language (version 4.5.1). Data analysis and chart construction were completed using GraphPad Prism 10. All data were presented as mean ± standard error of the mean (SEM). For data with a normal distribution, the unpaired two-tailed Student’s t-test was used for comparisons between two groups. Meanwhile, for multiple group comparisons with homogeneous variance, one-way analysis of variance followed by Tukey’s post hoc test was employed; in cases of heterogeneous variance, Kruskal-Wallis test with Dunn’s multiple comparison test was utilized. Two-way ANOVA was performed for comparisons of time-series differences, supplemented with Tukey’s post hoc test and Sidak’s post hoc test. For non-normally distributed data, the Mann-Whitney U test was employed. The threshold for statistical significance was set at *p* < 0.05.

## Results

### Elevated CMPK2 was identified during the development of NP

**Fig. 1:**
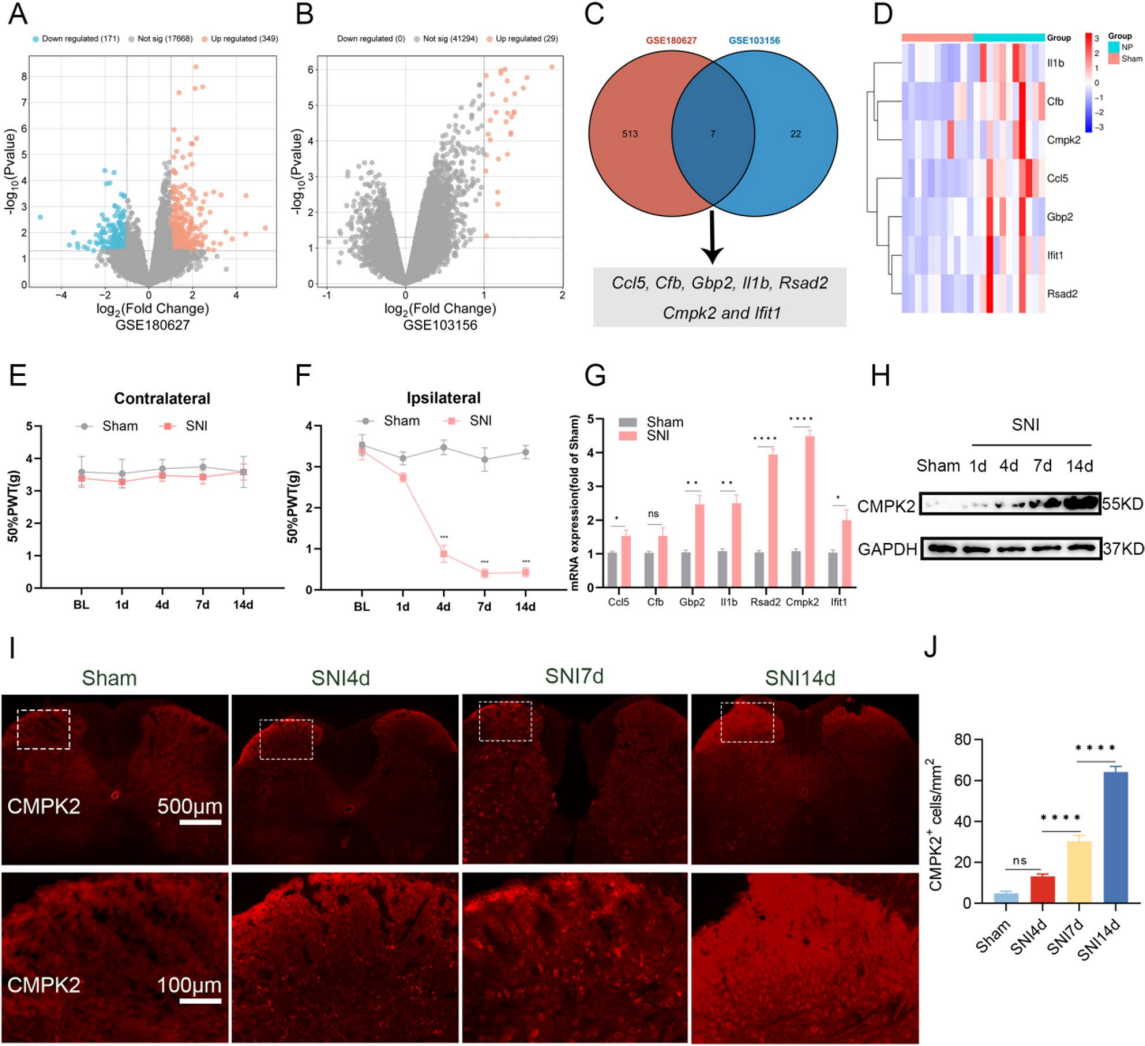
Identification of elevated CMPK2 during the development of NP. (**A**) Volcano plot showing DEGs in spinal microglia isolated from NP mice versus Sham controls (day 7 post-surgery) from the GSE180627 dataset, which includes samples from both sexes. (**B**) Volcano plot showing DEGs in BV2 microglial cells treated with LPS (1 µg/mL, 24 h) compared with untreated controls from the GSE103156 dataset. (**C**) Venn diagram illustrating the overlap of DEGs between the two datasets. (**D**) Heatmap displaying the expression profiles of the seven overlapping DEGs in spinal microglia from the GSE180627 dataset. (**E, F**) Behavioral assessment of mechanical allodynia using the PWT at baseline and on days 1, 4, 7, and 14 following SNI surgery (*n* = 6). (**G**) RT-qPCR validation of the expression of *Ccl5*, *Cfb*, *Gbp2*, *Il1b*, *Rsad2*, *Cmpk2* and *Ifit1* in SDH of SNI versus Sham mice (*n* = 6). (**H**) The protein level of CMPK2 in SDH after SNI by western blot (*n* = 6). (**I, J**) Representative immunofluorescence images showing CMPK2 expression in the SDH after SNI (Scale bars: 500 μm, 100 μm, *n* = 6). All data are presented as the mean ± SEM. ns, not significant; ^*^P < 0.05, ^**^P < 0.01, ^***^P < 0.001, ^****^P < 0.0001

To prioritize candidate genes consistently associated with microglial inflammatory activation across distinct experimental contexts, we performed a comparative transcriptomic analysis using both in vivo and in vitro datasets. We first analyzed the GSE180627 dataset, which contains spinal microglia isolated from NP and sham mice. Differential expression analysis identified 520 DEGs, including 349 upregulated and 171 downregulated genes (Fig. 1A). We then analyzed the GSE103156 dataset containing BV2 microglia stimulated with LPS (1 µg/mL, 24 h). A total of 29 upregulated DEGs were identified (Fig. 1B). Intersection of the two datasets yielded seven overlapping DEGs (Fig. 1C, D). To validate these bioinformatic findings, we established a SNI mouse model. Mechanical sensitivity was assessed using the von Frey test. As shown in Fig. 1E, F, the ipsilateral PWT in SNI mice was significantly reduced from day 1 to day 14, confirming successful induction of NP. No significant change was observed on the contralateral side.

Next, qRT-PCR was performed using SDH tissue to evaluate the expression of the seven candidate DEGs. All genes except *Cfb* were significantly upregulated in SNI mice relative to Sham controls, with *Cmpk2* showing the most pronounced increase (Fig. 1G). Based on these findings, we selected CMPK2 for further investigation. Western blotting (Fig. 1H) and immunofluorescence staining (Fig. 1I, J) demonstrated a time-dependent and sustained increase in CMPK2 protein expression in the spinal dorsal horn after SNI, with the highest level observed at day 14 post-injury. This temporal profile suggests that CMPK2 induction is not merely an acute response to nerve injury but is closely associated with the progression and maintenance of NP.

### Single-cell RNA sequencing and immunofluorescence reveal CMPK2 expression across multiple spinal cord cell types

**Fig. 2:**
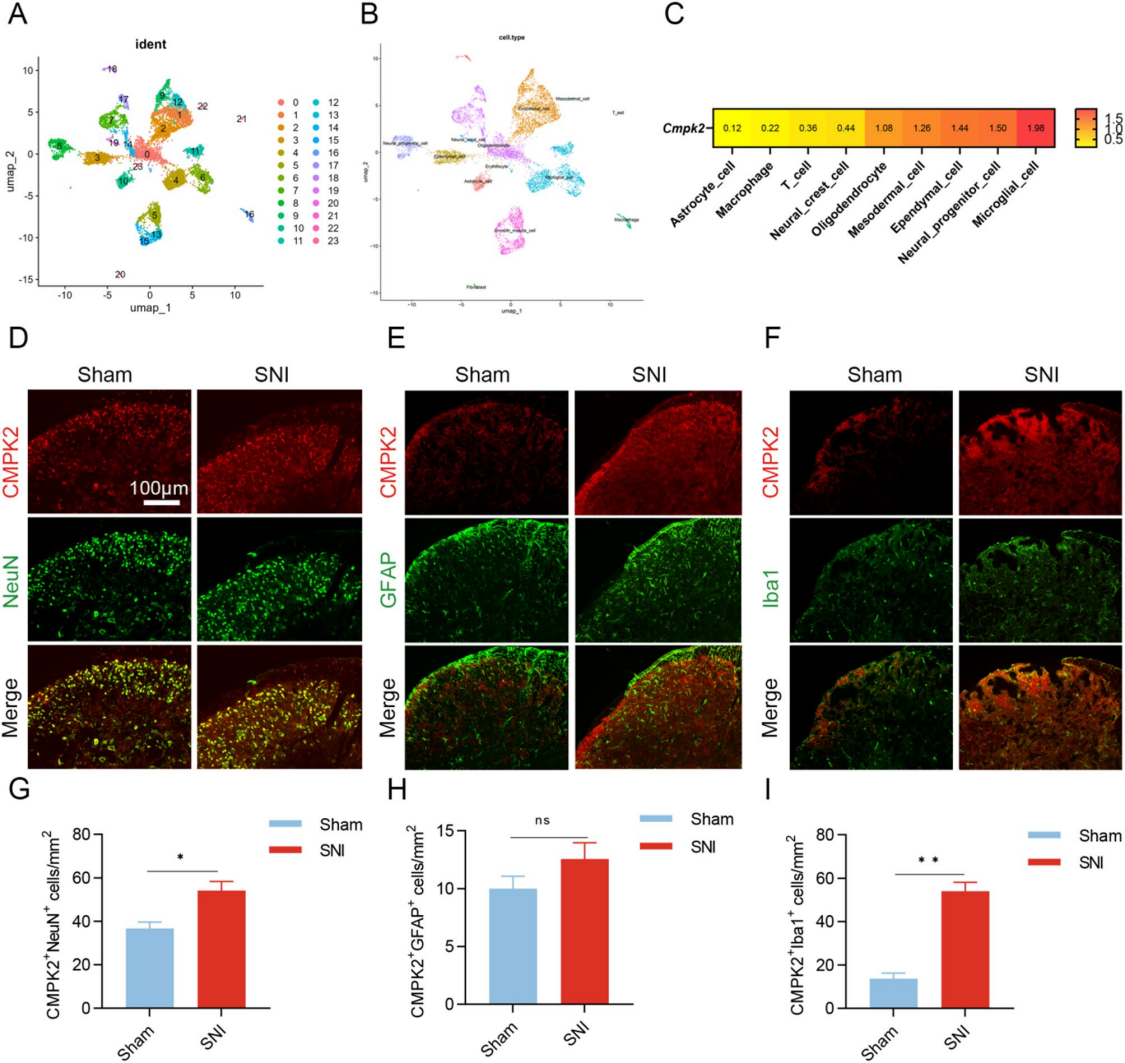
Single-cell RNA sequencing and immunofluorescence reveal CMPK2 expression across multiple spinal cord cell types. (**A-C**) Uniform manifold approximation and projection (UMAP) visualization of spinal cord single-cell RNA-seq data (GSE208766) with annotated cell clusters. The heatmap depicts the relative expression of *Cmpk2* across major spinal cord cell populations, highlighting its distribution among distinct cell types. (**D**) Representative double immunofluorescence staining of CMPK2 and NeuN in the SDH of Sham mice (scale bar = 100 μm, *n* = 6). (**E**) Representative double immunofluorescence staining of CMPK2 and GFAP in the SDH of SNI mice (scale bar = 100 μm, *n* = 6). (**F**) Representative double immunofluorescence staining of CMPK2 and Iba1 in the SDH of Sham mice (scale bar = 100 μm, *n* = 6). Images shown are representative of staining patterns observed across multiple sections and animals. (**G-H**) Quantitative analysis of CMPK2 co-localization with NeuN, GFAP and Iba-1 in Sham and SNI mice (*n* = 6). All data are presented as the mean ± SEM. ns, not significant; ^*^P < 0.05, ^**^P < 0.01

To characterize CMPK2 expression across major spinal cord cell types, we analyzed a single-cell RNA sequencing dataset (GSE208766). CMPK2 transcripts were detected in multiple spinal cord cell populations, with enrichment observed in microglia (Fig. 2A-C). To validate these findings in vivo, immunofluorescence staining was performed on SDH sections following SNI. CMPK2 immunoreactivity exhibited increased colocalization with both the microglial marker Iba1 and the neuronal marker NeuN after nerve injury, whereas no apparent change in colocalization with the astrocytic marker GFAP was observed (Fig. 2D-I). Collectively, these data demonstrate that CMPK2 is expressed in multiple spinal cord cell types under neuropathic conditions. Notably, the prominent enrichment of CMPK2 in microglia, together with its marked upregulation after SNI, suggests that microglia constitute a major cellular source contributing to CMPK2 elevation in the injured spinal cord.

### Cytosolic mtDNA accumulation and activation of the cGAS-STING pathway in SNI mice

**Fig. 3:**
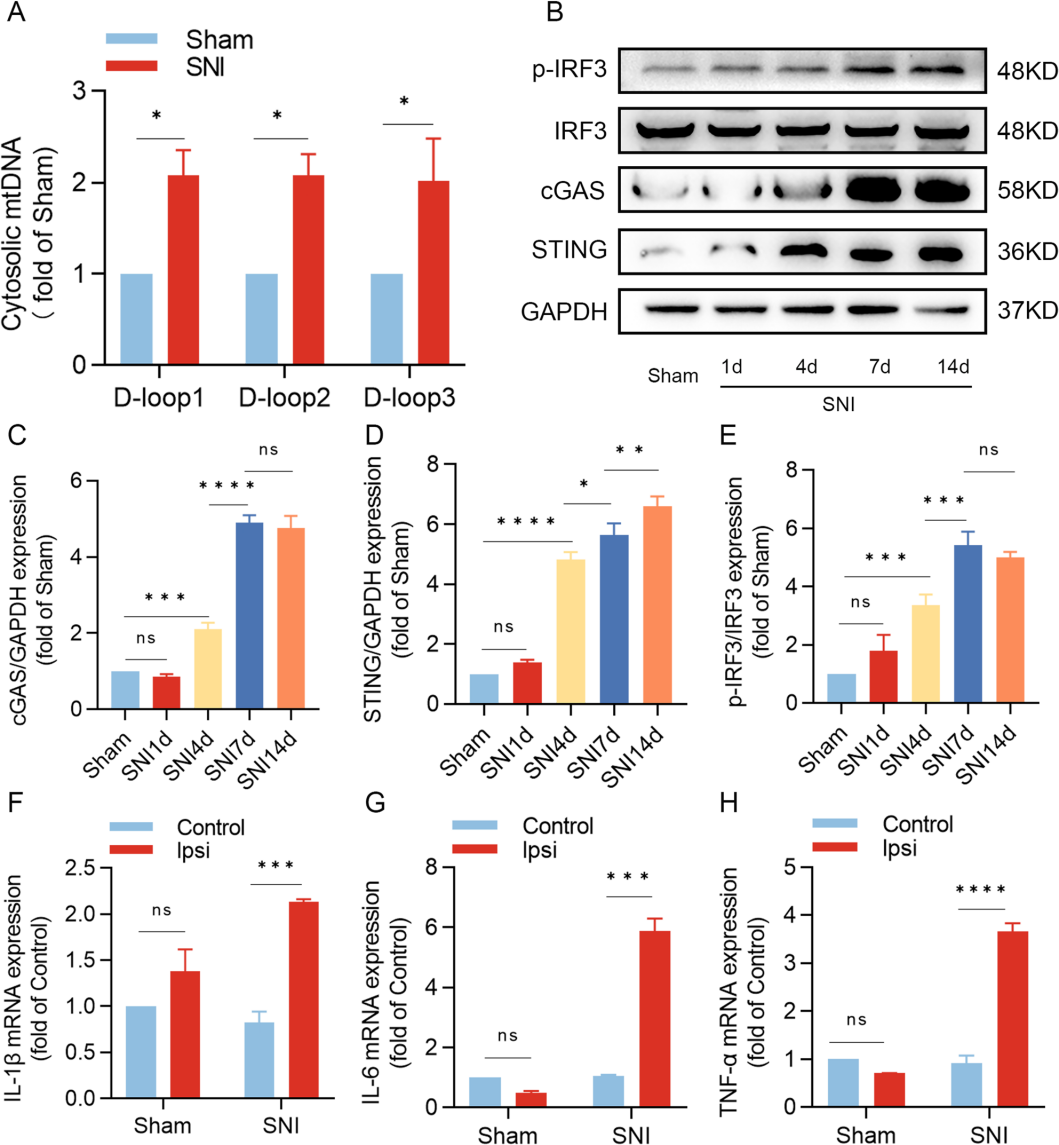
Cytosolic mtDNA release and activation of the cGAS-STING pathway in SNI mice. (**A**) Cytosolic mtDNA D-loops levels in SDH of Sham and SNI mice by RT-qPCR (*n* = 6). (**B-E**) Western blots and quantitative analysis of cGas, STING and p-IRF3 levels in SDH of Sham and SNI mice (*n* = 6). (**F-H**) Quantitative analysis of *IL-1β*, *TNF-α* and *IL-6* levels in SDH of Sham and SNI mice by RT-qPCR (*n* = 6). Ipsi, ipsilateral to the site of nerve injury; Contra, contralateral side. All data are presented as the mean ± SEM. ns, not significant; ^*^P < 0.05, ^**^P < 0.01, ^***^P < 0.001, ^****^P < 0.0001

Because CMPK2 is a key enzyme required for mitochondrial DNA (mtDNA) synthesis and has been implicated in promoting mtDNA release [16, 31], we assessed cytosolic mtDNA levels in the SDH of SNI and Sham mice. Cytosolic mtDNA, quantified using the D-loop levels, was significantly increased in SNI mice compared with Sham controls (Fig. 3A). Given that cytosolic mtDNA has been shown in recent studies to activate the cGAS-STING pathway, we measured its target protein levels in SDH tissues from both groups using Western blotting [34, 35]. The results indicated significantly higher protein levels of cGAS, STING, and p-IRF3 in the SNI group relative to the Sham group (Fig. 3B-E). Consistent with activation of innate immune signaling, qRT-PCR analysis showed significant increases in the pro-inflammatory cytokines *IL-1β*, *TNF-α*, and *IL-6* in SNI mice relative to Sham controls (Fig. 3F-H). These findings indicate that SNI induces cytosolic mtDNA accumulation and subsequent activation of the cGAS-STING-IRF3 inflammatory cascade.

### CMPK2 downregulation reduces cytosolic mtDNA accumulation, suppresses cGAS-STING signaling, and alleviates mechanical allodynia in vivo

**Fig. 4:**
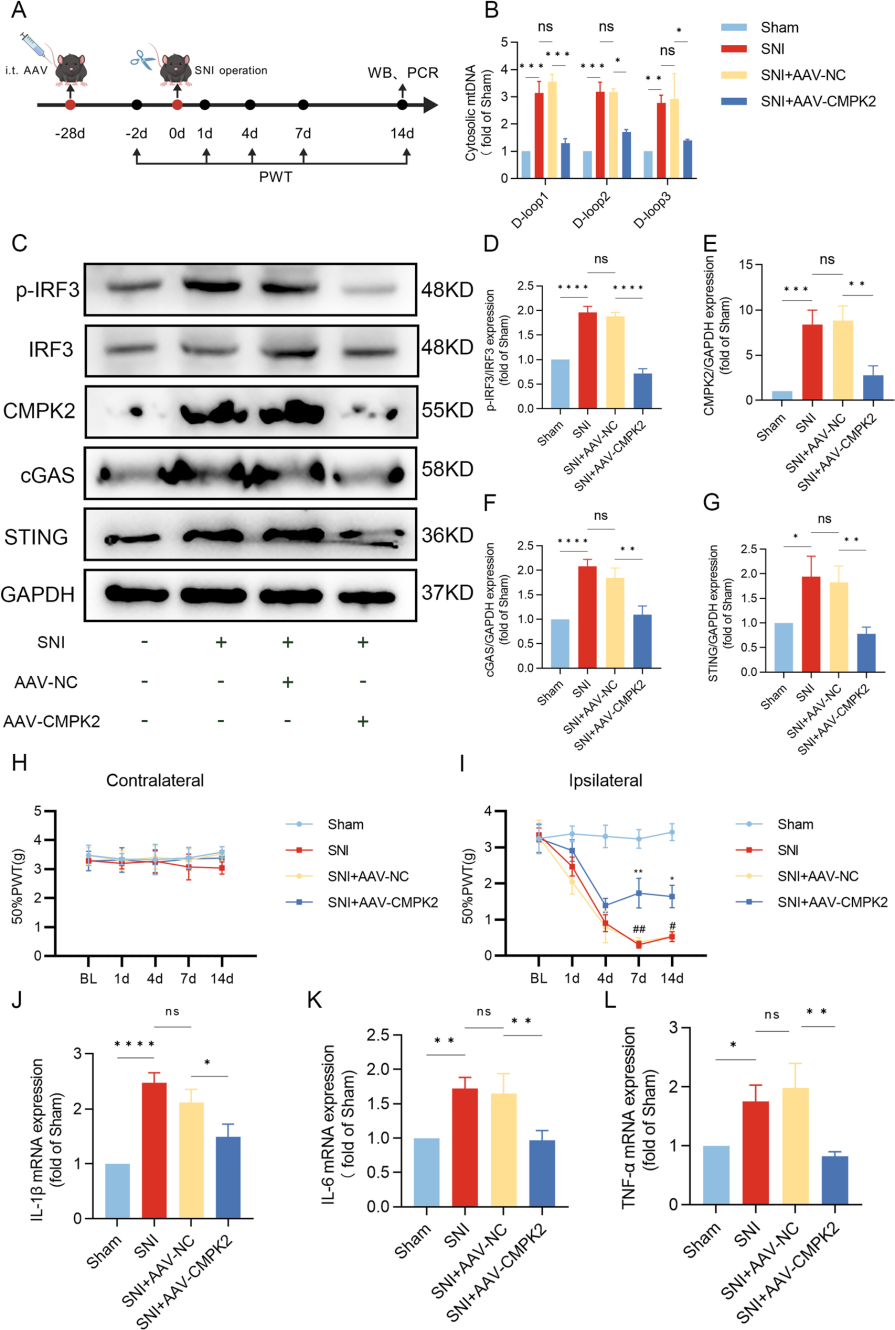
Downregulation of CMPK2 inhibits cytosolic mtDNA release, suppresses cGAS-STING activation, and alleviates mechanical allodynia in vivo. (**A**) Schematic illustration of the experimental timeline in which mice received intrathecal AAV-*Cmpk2* or AAV-NC injection 28 days prior to SNI surgery. (**B**) Cytosolic mtDNA D-loops levels in SDH of Sham, SNI, SNI + AAV-NC and SNI + AAV-*Cmpk2* mice by RT-qPCR (*n* = 6). (**C-G**) Western blots and quantitative analysis of CMPK2, cGas, STING and p-IRF3 levels in SDH of Sham, SNI, SNI + AAV-NC and SNI + AAV-*Cmpk2* mice (*n* = 6). (**H, I**) Mechanical allodynia evaluated by the PWT at baseline, 1, 4, 7 and 14 days from Sham, SNI, SNI + AAV-NC and SNI + AAV-*Cmpk2* mice (*n* = 6). (**J-L**) Quantitative analysis of *IL-1β*, *TNF-α* and *IL-6* levels in SDH of Sham, SNI, SNI + AAV-NC and SNI + AAV-*Cmpk2* mice by RT-qPCR (*n* = 6). All data are presented as the mean ± SEM. ns, not significant; ^*^P < 0.05, ^#^*P* < 0.05, ^**^P < 0.01, ^##^*P* < 0.01, ^***^P < 0.001, ^****^P < 0.0001

To examine the functional role of CMPK2 in NP, an adeno-associated virus expressing *Cmpk2* shRNA (AAV-*Cmpk2*) was intrathecally administered 28 days before SNI induction (Fig. 4A). As shown in Fig. 4C and D, CMPK2 expression in SDH was markedly reduced in the SNI + AAV-*Cmpk2* group compared with both the SNI and SNI + AAV-NC groups, confirming effective AAV-mediated knockdown.

We next quantified cytosolic mtDNA levels in SDH using the D-loop levels as an indicator. Cytosolic mtDNA accumulation was significantly decreased in the SNI + AAV-*Cmpk2* group relative to the SNI and SNI + AAV-NC groups (Fig. 4B). Western blot analysis further demonstrated that the protein levels of cGAS, STING, and phosphorylated IRF3 (p-IRF3) were markedly reduced following CMPK2 knockdown (Fig. 4C-G), indicating suppression of the cGAS-STING pathway. Behaviorally, the von Frey test revealed that AAV-mediated CMPK2 knockdown significantly attenuated mechanical allodynia in the ipsilateral hind paw, whereas contralateral responses remained unchanged (Fig. 4H-I).

Finally, qRT-PCR analysis showed that the expression of the pro-inflammatory cytokines *IL-1β*, *TNF-α*, and *IL-6* was significantly lower in the SNI + AAV-*Cmpk2* group compared with the SNI and SNI + AAV-NC groups (Fig. 4J-L). Together, these results indicate that CMPK2 contributes to NP pathogenesis by promoting cytosolic mtDNA accumulation and activation of cGAS-STING-IRF3 signaling, and that its knockdown alleviates both neuroinflammation and mechanical hypersensitivity.

### CMPK2 upregulation, mitochondrial dysfunction, cytosolic mtDNA accumulation, and activation of the cGAS-STING pathway in LPS-stimulated BV2 microglia

**Fig. 5:**
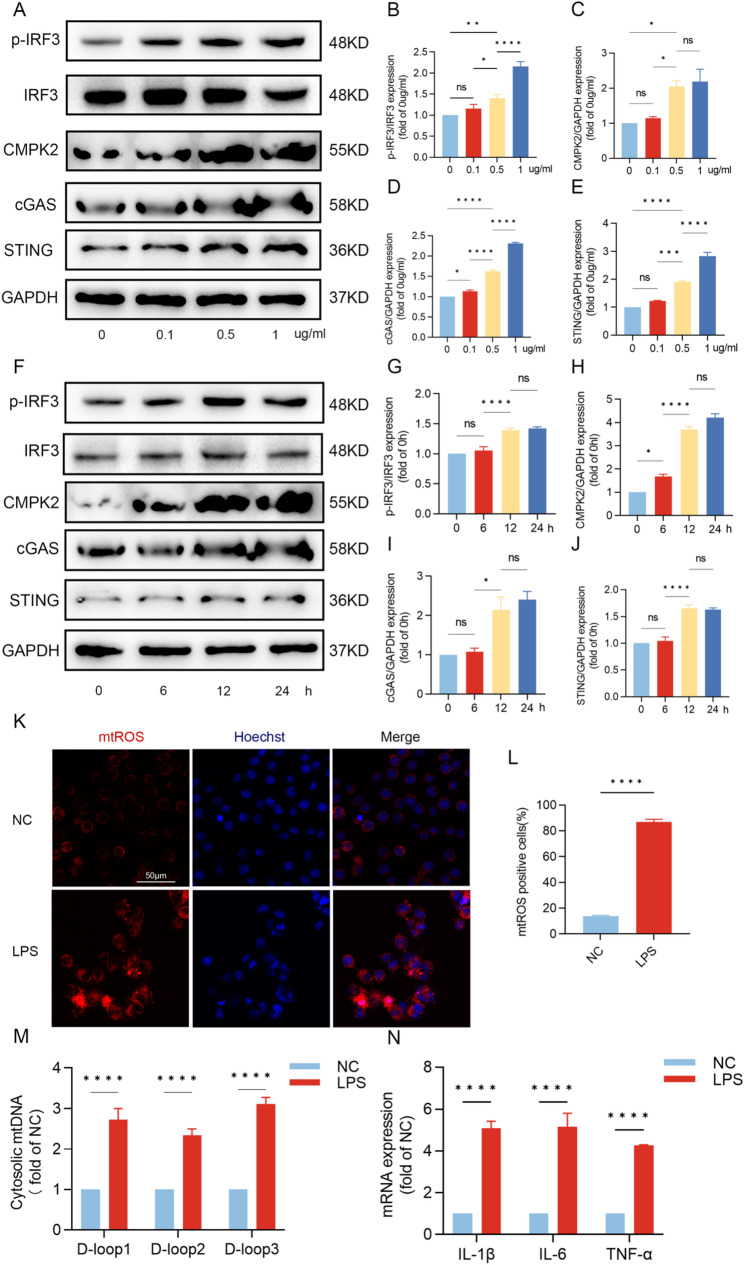
LPS induces CMPK2 upregulation, cytosolic mtDNA release, and activation of the cGAS-STING pathway in BV2 microglia. (**A-E**) Western blots and quantitative analysis of CMPK2, cGas, STING and p-IRF3 levels in LPS-induced BV2 microglia of 0, 0.1, 0.5 and 1 µg/mL after 24 h (*n* = 3). (**F-J**) Western blots and quantitative analysis of CMPK2, cGas, STING and p-IRF3 levels in LPS-induced BV2 microglia of 1 µg/mL after 0, 6, 12 and 24 h (*n* = 3). (**K, L**) Representative of immunofluorescence staining of MitoSOX, a mitochondria-targeted superoxide probe, in LPS (1 µg/mL)-induced BV2 microglia after 24 h and quantitative analysis of mtROS levels (scale bars: 50 μm). (**M**) Cytosolic mtDNA D-loops levels in LPS (1 µg/mL)-induced BV2 microglia after 24 h by RT-qPCR (*n* = 3). (**N**) Quantitative analysis of *IL-1β*, *TNF-α* and *IL-6* levels in LPS (1 µg/mL)-induced BV2 microglia after 24 h by RT-qPCR (*n* = 3). All data are presented as the mean ± SEM. ns, not significant; ^*^P < 0.05, ^**^P < 0.01, ^***^P < 0.001, ^****^P < 0.0001

To further explore the association between CMPK2 and microglial inflammatory activation, BV2 microglia were stimulated with LPS. Western blot analysis showed that LPS treatment increased the protein levels of CMPK2, cGAS, STING, and p-IRF3 in both dose- and time-dependent manners (Fig. 5A-J). Mitochondrial reactive oxygen species (mtROS) were assessed using MitoSOX Red staining. LPS stimulation led to elevated mtROS levels, indicating impaired mitochondrial function (Fig. 5K-L). Consistent with mitochondrial stress, cytosolic mtDNA quantified using the D-loop levels was significantly increased following LPS exposure (Fig. 5M). Finally, qRT-PCR analysis showed that LPS stimulation markedly upregulated the pro-inflammatory cytokines *IL-1β*, *TNF-α*, and *IL-6* in BV2 microglia (Fig. 5N). These findings demonstrate that LPS-induced microglial activation is accompanied by CMPK2 upregulation, mitochondrial oxidative stress, cytosolic mtDNA accumulation, and activation of the cGAS-STING-IRF3 inflammatory pathway.

### CMPK2 knockdown attenuates mtDNA release and suppresses activation of the cGAS-STING pathway in LPS-stimulated BV2 microglia

**Fig. 6:**
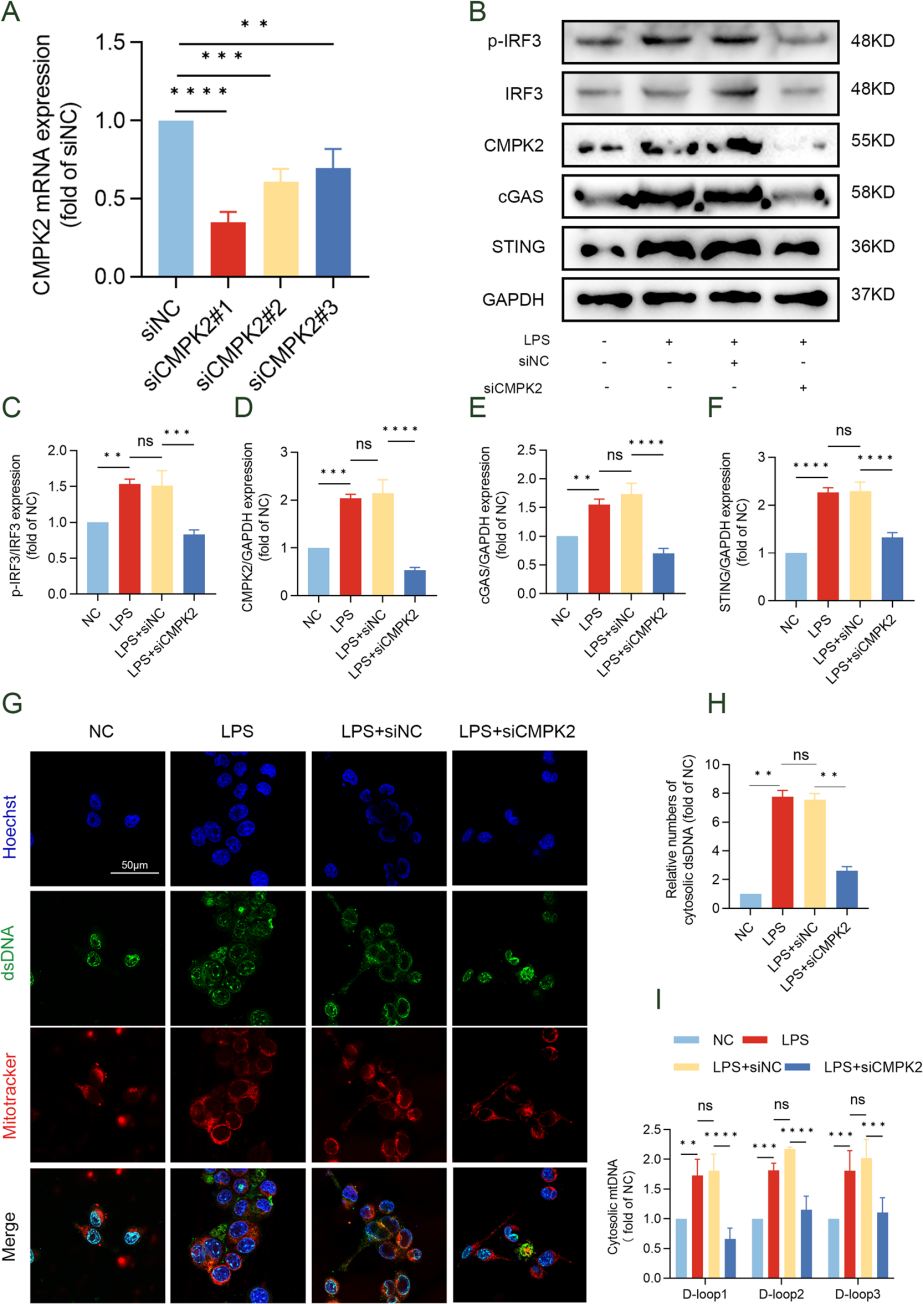
CMPK2 promotes mtDNA cytosolic release and activation of the cGAS-STING pathway in LPS-treated BV2 microglia. (**A**) RT-qPCR analysis confirming the efficiency of *Cmpk2* knockdown in BV2 microglia transfected with si-*Cmpk2* or scrambled control (si-NC) following LPS stimulation (*n* = 3). (**B-F**) Western blots and quantitative analysis of CMPK2, cGas, STING and p-IRF3 levels in microglia of Normal control (NC), LPS, LPS + si-NC and LPS + si-*Cmpk2* (*n* = 3). (**G**) Representative of immunofluorescence staining of Mitotracker and dsDNA in microglia of NC, LPS, LPS + si-NC and LPS + si-*Cmpk2* (Scale bar: 50 μm, *n* = 3). (**H**) Relative numbers of cytosolic dsDNA in microglia of NC, LPS, LPS + si-NC and LPS + si-*Cmpk2* (*n* = 3). (**I**) Cytosolic mtDNA D-loops levels in microglia of NC, LPS, LPS + si-NC and LPS + si-*Cmpk2* by RT-qPCR (*n* = 3). All data are presented as the mean ± SEM. ns, not significant; ^**^P < 0.01, ^***^P < 0.001, ^****^P < 0.0001

To further examine the role of CMPK2 in regulating mtDNA release and downstream signaling, *Cmpk2* expression in BV2 microglia was silenced using siRNA. As shown in Fig. 6A, siRNA treatment effectively reduced *Cmpk2* expression. Correspondingly, knockdown of *Cmpk2* markedly decreased the protein levels of CMPK2, cGAS, STING, and p-IRF3 in LPS-stimulated cells (Fig. 6B-F). Immunofluorescence staining was used to visualize cytosolic DNA localization. Double-stranded DNA (dsDNA) signals showed minimal overlap with Hoechst-labeled nuclei or MitoTracker-labeled mitochondria and were instead distributed in the cytosol (Fig. 6G). Quantification revealed that LPS markedly increased cytosolic dsDNA levels, whereas *Cmpk2* knockdown significantly attenuated this increase (Fig. 6H). To further determine whether the cytosolic dsDNA originated from mitochondria, we measured cytosolic mtDNA using the D-loop levels. siRNA-mediated *Cmpk2* silencing significantly reduced cytosolic mtDNA accumulation in LPS-treated BV2 microglia (Fig. 6I). Collectively, these findings indicate that CMPK2 contributes to LPS-induced cytosolic mtDNA accumulation and the subsequent activation of the cGAS-STING-IRF3 signaling pathway in microglia.

### CMPK2 knockdown attenuates mtDNA release and preserves mitochondrial function in primary microglia

**Fig. 7:**
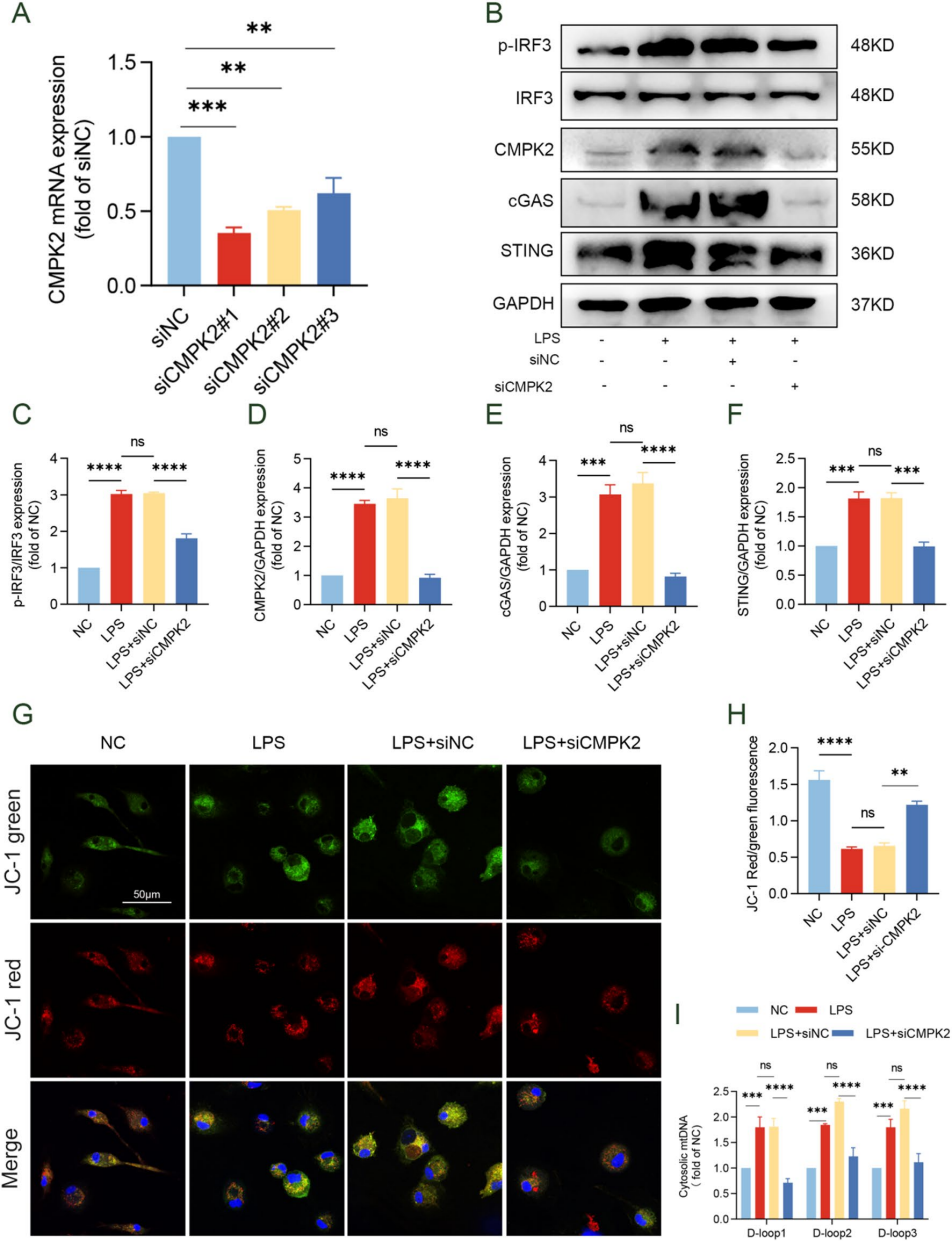
CMPK2 knockdown attenuates mtDNA release and preserves mitochondrial function in primary microglia. (**A**) RT-qPCR analysis showing knockdown efficiency of *Cmpk2* in primary microglia following si-*Cmpk2* transfection (*n* = 3). (**B-F**) Representative immunoblots and quantitative analyses of CMPK2, cGAS, STING, and p-IRF3 protein levels in primary microglia under the indicated conditions (*n* = 3, NC, LPS, LPS + siNC, and LPS + si-CMPK2). (**G**) Representative JC-1 staining images showing mitochondrial membrane potential in primary microglia (scale bar = 20 μm, *n* = 3). (**H**) Quantification of mitochondrial membrane potential (ΔΨm) expressed as the ratio of red to green fluorescence intensity (*n* = 3). (**I**) Cytosolic mtDNA release assessed by RT-qPCR detection of mitochondrial D-loop sequences in primary microglia under the indicated conditions (*n* = 3). All data are presented as the mean ± SEM. ns, not significant; ^**^P < 0.01, ^***^P < 0.001, ^****^P < 0.0001

To validate the physiological relevance of CMPK2-dependent mitochondrial dysfunction and innate immune activation beyond immortalized microglial cell lines, we next performed mechanistic analyses in primary mouse microglia. Primary microglia were transfected with siRNA targeting *Cmpk2* (si-CMPK2) or a scrambled control (siNC) and subsequently stimulated with LPS (1 µg/mL) for 24 h. RT-qPCR analysis confirmed efficient knockdown of *Cmpk2* expression in primary microglia following si-*Cmpk2* transfection compared with si-NC controls (Fig. 7A). Consistent with the reduced expression of CMPK2, immunoblot analyses revealed that LPS-induced upregulation of CMPK2 was markedly attenuated by *Cmpk2* knockdown. Moreover, silencing of *Cmpk2* significantly suppressed the activation of the cGAS-STING-IRF3 pathway, as evidenced by decreased protein levels of cGAS, STING, and p-IRF3, compared with LPS-treated and si-NC-transfected cells (Fig. 7B-F). Given the established role of CMPK2 in regulating mitochondrial integrity, we next assessed mitochondrial function in primary microglia. JC-1 staining demonstrated that LPS stimulation induced a pronounced loss of mitochondrial membrane potential (ΔΨm), characterized by a reduced red-to-green fluorescence ratio, whereas *Cmpk2* knockdown largely preserved mitochondrial membrane potential under inflammatory conditions (Fig. 7G, H). Consistent with the preservation of mitochondrial function, cytosolic mtDNA release was significantly increased in LPS-stimulated primary microglia, as measured by RT-qPCR detection of mitochondrial D-loop sequences. Importantly, *Cmpk2* knockdown markedly reduced cytosolic mtDNA accumulation compared with LPS and LPS + siNC groups (Fig. 7I). Collectively, these data demonstrate that CMPK2 knockdown in primary microglia attenuates mitochondrial dysfunction, limits mtDNA release, and suppresses cGAS-STING-IRF3 signaling under inflammatory stimulation, thereby corroborating our mechanistic findings obtained in BV2 microglia.

### Exogenous mtDNA reverses the effects of CMPK2 knockdown, and STING inhibition blocks mtDNA-induced signaling activation

**Fig. 8:**
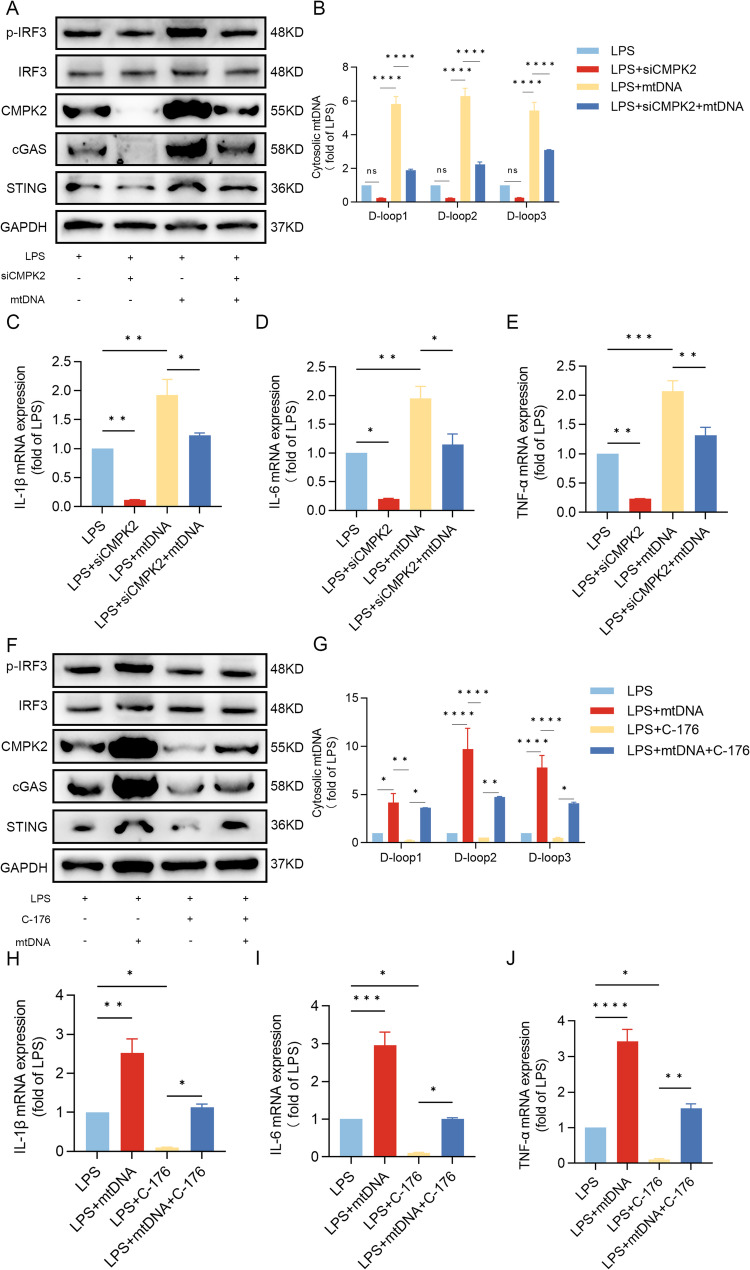
CMPK2-mediated mtDNA release-induced activation of the cGAS-STING pathway can be rescued by mtDNA supplementation or STING inhibition in LPS-treated BV2 microglia. (**A**) Western blot analysis of cGAS, STING, and p-IRF3 in LPS-treated (1 µg/ml) BV2 microglia transfected with si-*Cmpk2* and/or exogenous mtDNA. Groups include: LPS, LPS + si-*Cmpk2*, LPS + mtDNA, and LPS + si-*Cmpk2* + mtDNA (*n* = 3). (**B**) RT-qPCR quantification of cytosolic mtDNA D-loop levels in the above four treatment groups (*n* = 3). (**C-E**) RT-qPCR analysis of *Il-1β*, *TNF-α*, and *Il-6* mRNA levels in microglia from the four groups (*n* = 3). (**F**) Western blot analysis of cGAS, STING, and p-IRF3 in BV2 microglia transfected with mtDNA and treated with the STING inhibitor C-176. Groups include: LPS, LPS + mtDNA, LPS + C-176, and LPS + mtDNA + C-176 (*n* = 3). (**G**) RT-qPCR quantification of cytosolic mtDNA D-loop levels in the four groups (*n* = 3). (**H-J**) RT-qPCR analysis of *Il-1β*, *TNF-α*, and *Il-6* expression in microglia from the four groups treated with mtDNA and/or C-176 (*n* = 3). All data are presented as the mean ± SEM. ns, not significant; ^*^P < 0.05, ^**^P < 0.01, ^***^P < 0.001, ^****^P < 0.0001

To determine whether the effects of CMPK2 on cGAS-STING signaling are mediated by cytosolic mtDNA, we performed rescue experiments in BV2 microglia. LPS-treated cells were co-transfected with si-*Cmpk2* and exogenous mtDNA. Western blot analysis showed that exogenous mtDNA restored the levels of cGAS, STING, and p-IRF3, which were reduced by *Cmpk2* knockdown (Fig. 8A and Fig. S1A-D). Consistently, cytosolic mtDNA levels and the mRNA expression of *IL-1β*, *TNF-α*, and *IL-6* were also rescued following mtDNA supplementation (Fig. 8B-E). In a complementary experiment, LPS-treated BV2 cells were transfected with mtDNA and subsequently treated with C-176, a selective STING inhibitor. C-176 treatment prevented mtDNA-induced activation of STING and p-IRF3 and reduced downstream cytokine expression, while cytosolic mtDNA levels remained unaffected (Fig. 8F-J and Fig. S1E-H). These findings indicate that CMPK2 regulates inflammatory activation primarily through promoting cytosolic mtDNA accumulation, which subsequently drives cGAS-STING-IRF3 signaling in microglia.

### IRF3 contributes to transcriptional regulation of CMPK2

**Fig. 9:**
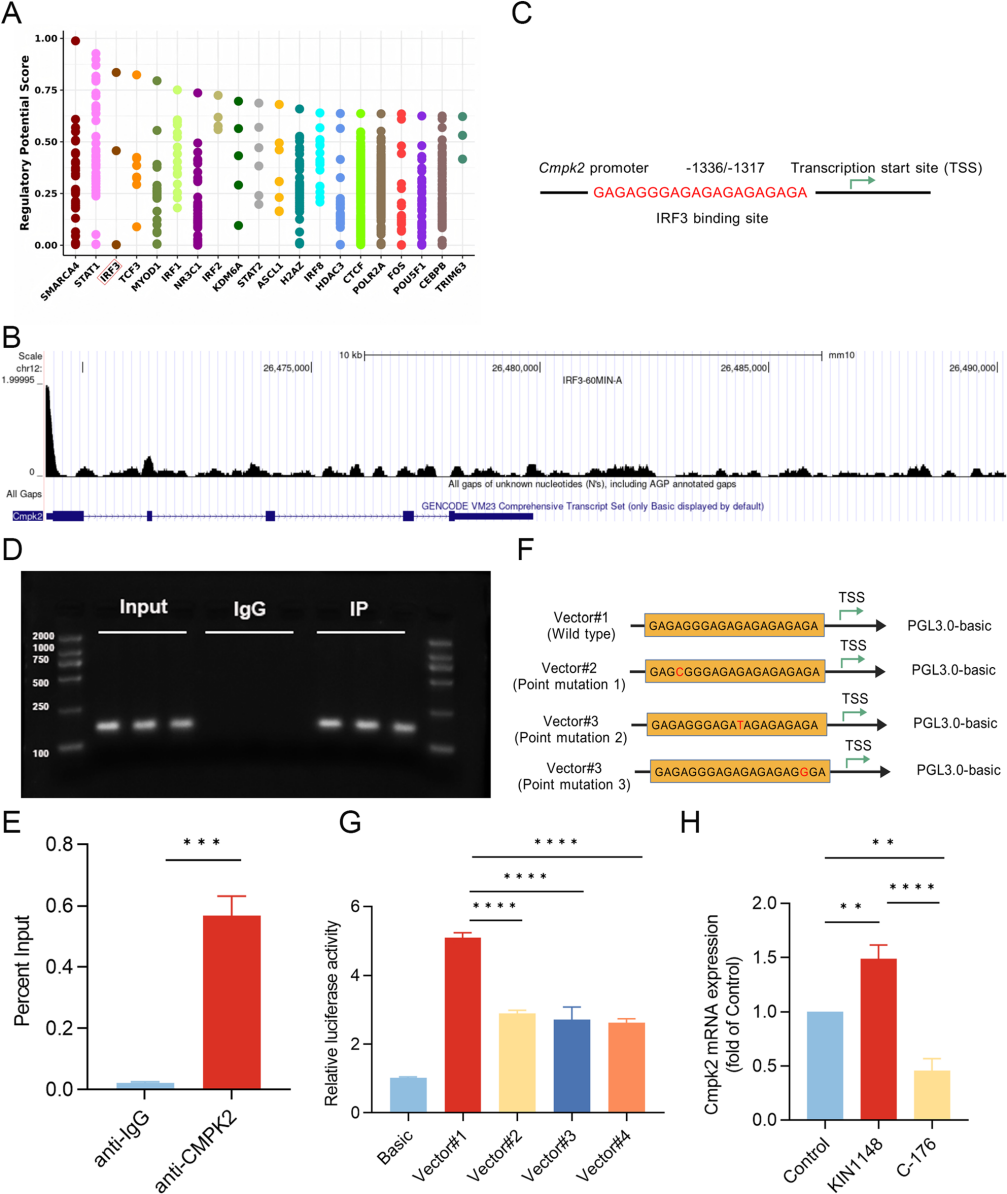
IRF3 enhances *Cmpk2* transcription by directly binding to its promoter. (**A**) Predicted transcription factor binding scores for the *Cmpk2* promoter, identifying candidate regulators of *Cmpk2* expression. (**B**) Analysis of IRF3 binding sites within the CMPK2 genomic locus based on publicly available ChIP-seq datasets from macrophages in the UCSC Genome Browser. (**C**) Predicted IRF3 binding motifs within the mouse *Cmpk2* promoter region identified using the AnimalTFDB v4.0 database. (**D, E**) ChIP-qPCR validation of IRF3 binding to the predicted promoter regions in BV-2 microglia using anti-IRF3 antibody or IgG control (*n* = 3). (**F**) Schematic representation of the wild-type *Cmpk2* promoter fragment (− 1336 to − 1317 bp) and the corresponding mutant constructs used for luciferase assays. (**G**) Dual-luciferase reporter assay showing the transcriptional activities of wild-type and mutant *Cmpk2* promoters in BV-2 microglia stimulated with LPS (1 µg/mL). (*n* = 3). (**H**) RT-qPCR analysis of *Cmpk2* mRNA levels in BV2 microglia treated with Control, the IRF3 agonist KIN1148, or the STING inhibitor C-176 (*n* = 3). All data are presented as the mean ± SEM. ^**^P < 0.01, ^***^P < 0.001, ^****^P < 0.0001

To identify transcriptional regulators associated with CMPK2 upregulation during NP, we first predicted potential CMPK2-associated transcription factors using the Cistrome Data Browser. IRF3 appeared among the top candidates (Fig. 9A). Consistently, public ChIP-seq data from the UCSC Genome Browser suggested a putative IRF3-binding region within the *Cmpk2* promoter (Fig. 9B). Further prediction using AnimalTFDB v4.0 revealed multiple IRF family consensus motifs in the mouse *Cmpk2* promoter (Fig. 9C). We next performed ChIP-qPCR in BV2 microglia, which indicated that IRF3 is associated with the *Cmpk2* promoter region under inflammatory stimulation (Fig. 9D, E). To assess whether IRF3 influences *Cmpk2* transcriptional activity, we constructed wild-type and IRF3-binding-site–mutant *Cmpk2* promoter luciferase reporters. Dual-luciferase assays showed that LPS markedly increased wild-type promoter activity, whereas mutation of the predicted IRF3-binding site attenuated this LPS-induced activation (Fig. 9F, G). Finally, pharmacological modulation of IRF3 further supported this regulatory relationship. Treatment with the IRF3 agonist KIN1148 increased *Cmpk2* mRNA expression in BV2 microglia, whereas C-176, a STING inhibitor known to suppress IRF3 activation, reduced *Cmpk2* expression (Fig. 9H). Together, these findings suggest that IRF3 may contribute to the transcriptional regulation of *Cmpk2* in microglial cells under inflammatory conditions.

### Integrated computational and experimental analyses support direct interaction between NDGA and CMPK2

**Fig. 10:**
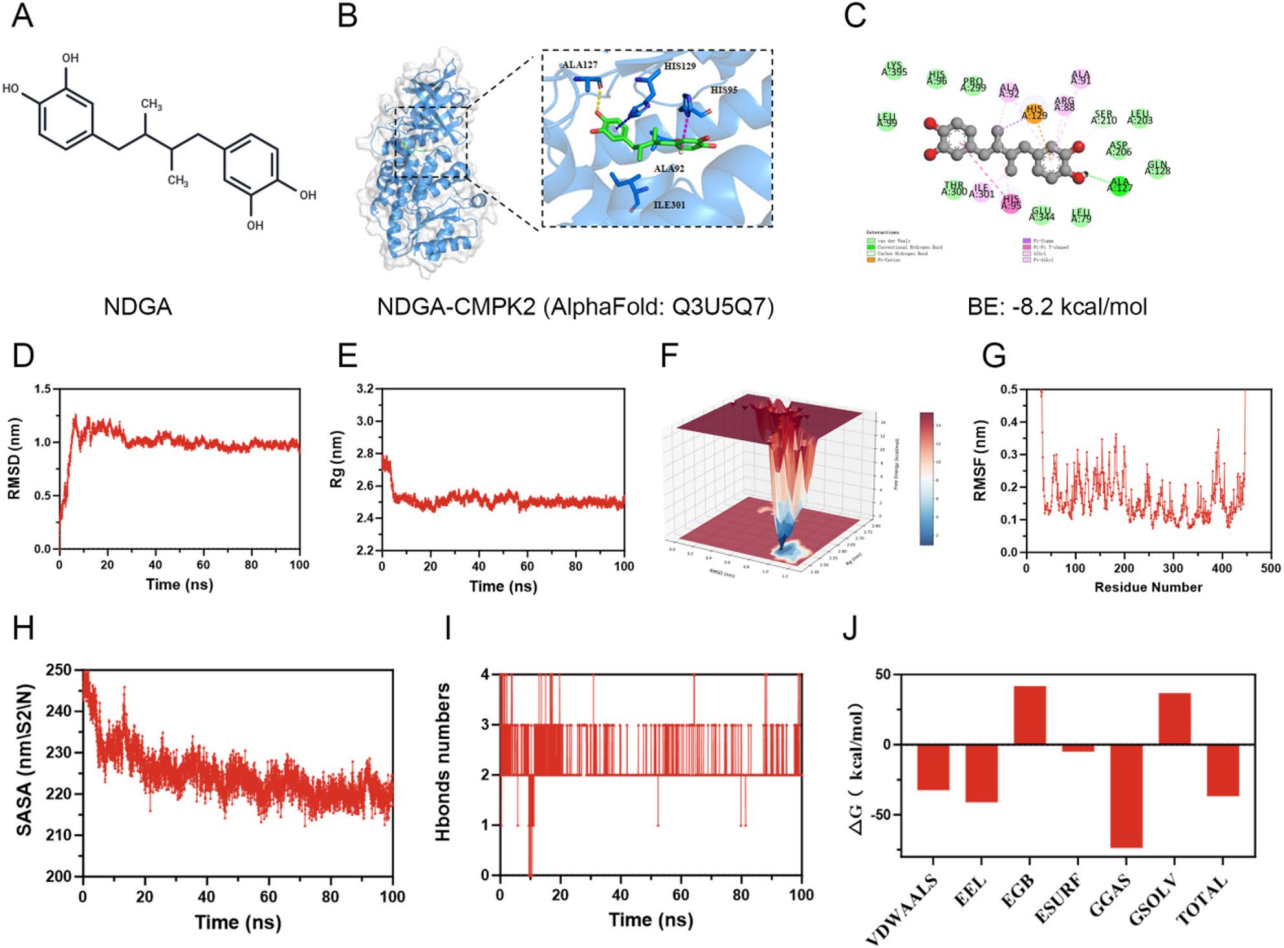
Integrated computational and experimental analyses of NDGA–CMPK2 interaction. (**A**) Chemical structure of NDGA. (**B-C**) Molecular docking of NDGA within the CMPK2 binding pocket, showing predicted binding modes and docking score. (**D**) Root mean square deviation (RMSD) of the CMPK2-NDGA complex during the 100-ns molecular dynamics (MD) simulation. (**E**) Radius of gyration (Rg) of the protein-ligand complex over time. (**F**) Free energy landscape (FEL) constructed based on RMSD and Rg. (**G**) Root mean square fluctuation (RMSF) of CMPK2 residues. (**H**) Buried solvent-accessible surface area (SASA) of the complex during MD simulation. (**I**) Number of intermolecular hydrogen bonds between NDGA and CMPK2 over time. (**J**) Binding free energy of the CMPK2-NDGA complex calculated by MM/GBSA analysis

Given the role of CMPK2 in cytosolic mtDNA release and neuroinflammatory responses, we explored potential CMPK2-targeting compounds with relevance to NP. Previous studies have suggested NDGA as a candidate modulator of CMPK2. To evaluate its potential interaction with CMPK2, we first performed molecular docking using the CMPK2 crystal structure. NDGA was predicted to fit within the canonical binding pocket, with a docking score of − 8.2 kcal/mol, suggesting favorable binding (Fig. 10A-C).

To further assess the stability of this interaction, we conducted a 100-ns molecular dynamics (MD) simulation. The root-mean-square deviation (RMSD) of the protein-ligand complex showed an initial equilibration phase followed by stabilization at approximately 1.0 nm, indicating convergence to a stable configuration (Fig. 10D). The radius of gyration (Rg) remained stable at around 2.5 nm throughout the simulation (Fig. 10E), suggesting that ligand binding did not induce major conformational changes in CMPK2. Free-energy landscape (FEL) analysis revealed a predominant low-energy basin, consistent with a stable conformational state (Fig. 10F). Root-mean-square fluctuation (RMSF) analysis further indicated limited residue-level fluctuations, supporting structural stability around the binding interface (Fig. 10G).

Analysis of the protein-ligand interface showed that the buried solvent-accessible surface area (SASA) remained stable during the simulation (Fig. 10H), indicating persistent intermolecular contacts. In addition, NDGA maintained 2–3 hydrogen bonds with CMPK2 throughout the simulation (Fig. 10I), which may contribute to binding stability. MM/GBSA calculations further estimated a mean binding free energy of − 36.83 ± 2.85 kcal/mol (Fig. 10J), supporting a thermodynamically favorable interaction.

We next examined whether NDGA inhibits CMPK2 enzymatic activity. In vitro kinase assays showed that NDGA suppressed CMPK2 activity in a concentration-dependent manner, with an IC₅₀ value of 5.26 µM (Fig. S2A). To further assess target engagement in a cellular context, a CETSA was performed. Compared with DMSO-treated cells, NDGA treatment increased the thermal stability of CMPK2, supporting cellular engagement of CMPK2 by NDGA (Fig. S2B). Collectively, these findings, together with molecular docking and molecular dynamics analyses, support CMPK2 as a functionally relevant target of NDGA.

### NDGA attenuates cytosolic mtDNA release, cGAS-STING activation, and pain-related behaviors in SNI mice

**Fig. 11:**
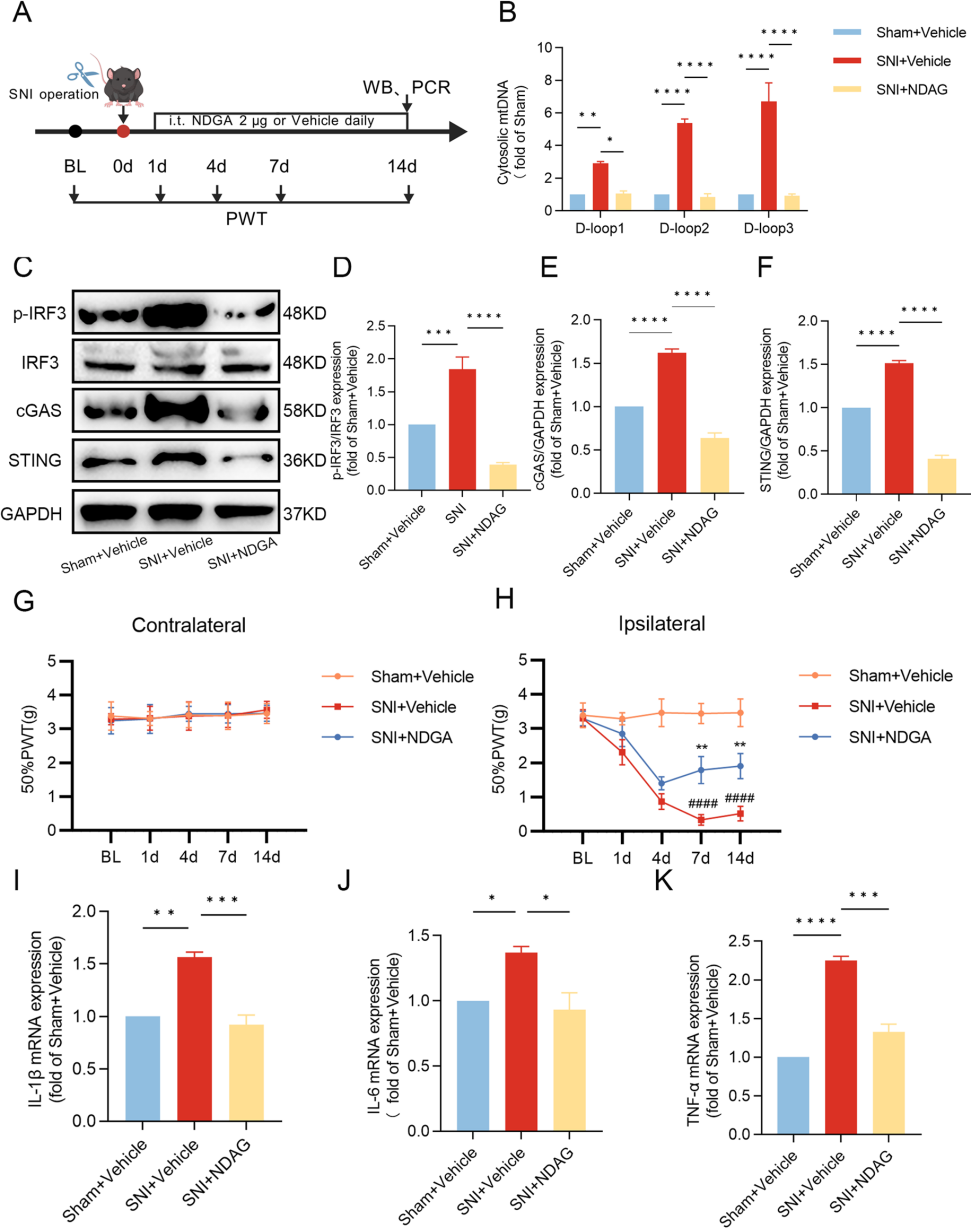
NDGA suppresses cytosolic mtDNA escape, inhibits cGAS-STING activation, and alleviates mechanical allodynia in SNI mice. (**A**) Schematic timeline illustrating NDGA administration in the SNI pain model. (**B**) Western blot and quantitative analyses of cGAS, STING, and p-IRF3 levels in SDH from Sham + Vehicle, SNI + Vehicle, and SNI + NDGA groups (*n* = 6). (**C**) Cytosolic mtDNA D-loops levels in SDH of Sham + Vehicle, SNI + Vehicle, SNI + NDGA group by RT-qPCR (*n* = 6). (**D**) Quantitative analysis of *IL-1β*, *TNF-α* and *IL-6* levels in SDH of Sham + Vehicle, SNI + Vehicle, SNI + NDGA group by RT-qPCR (*n* = 6). (**E**) Mechanical allodynia evaluated by the PWT at baseline, 1, 4, 7 and 14 days in the above three treatment groups (*n* = 6). All data are presented as the mean ± SEM. ns, not significant; ^*^P < 0.05, ^**^P < 0.01, ^***^P < 0.001, ^****^P < 0.0001, ^####^*P* < 0.0001

To explore the in vivo effects of NDGA in NP, SNI mice received intrathecal NDGA administration following nerve injury (Fig. 11A). NDGA treatment was associated with a reduction in cytosolic mtDNA D-loop copy numbers compared with vehicle-treated SNI mice (Fig. 11B). Western blot analysis further showed that NDGA attenuated the SNI-induced upregulation of cGAS, STING, and p-IRF3 in SDH tissues (Fig. 11C-F), suggesting modulation of the mtDNA-cGAS-STING signaling pathway. Behavioral assessment indicated that NDGA treatment attenuated mechanical allodynia in SNI mice at days 7 and 14 after administration (Fig. 11G, H), whereas no significant change was observed at day 4, potentially reflecting the time required for modulation of upstream molecular processes. In addition, NDGA treatment reduced spinal expression of pro-inflammatory cytokines, including IL-1β, TNF-α, and IL-6, compared with vehicle-treated SNI mice (Fig. 11I-K). Together, these findings indicate that post-injury NDGA administration modulates mtDNA release, innate immune signaling, and pain-related behaviors in vivo.

## Discussion

**Fig. 12:**
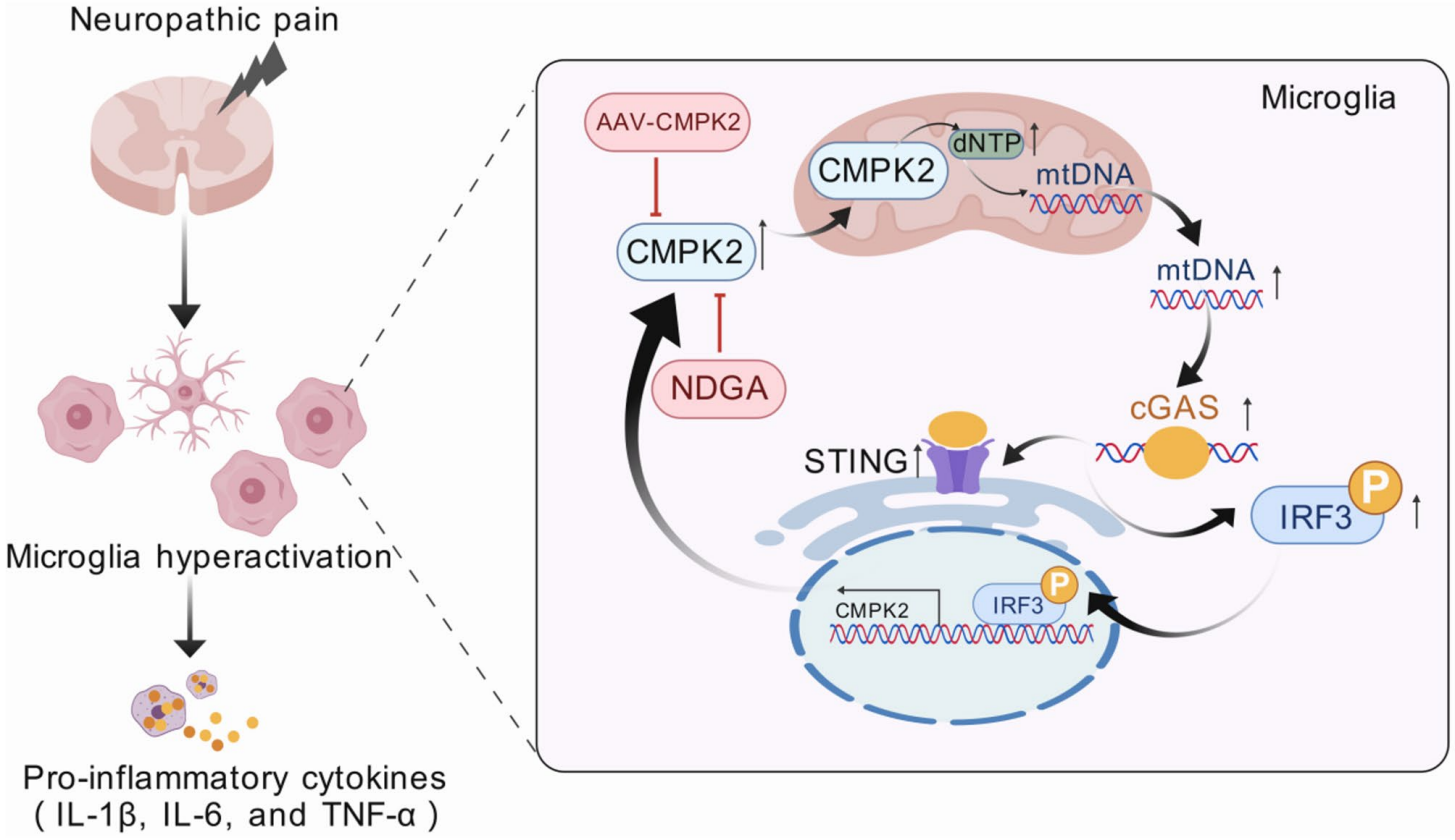
Schematic illustration of the role CMPK2 in neuropathic pain

In this study, we identify an immunometabolic mechanism through which mitochondrial dysfunction within the spinal cord microenvironment may contribute to sustained neuroinflammation and NP. Our data show that peripheral nerve injury induces mitochondrial damage in the spinal dorsal horn, accompanied by cytosolic release of mtDNA and activation of the cGAS-STING-IRF3 innate immune pathway. Although CMPK2 expression is detectable in multiple spinal cord cell types, including neurons, both single-cell RNA sequencing and immunofluorescence analyses indicate that microglia represent a major cellular population exhibiting elevated CMPK2 expression following nerve injury. Within this multicellular context, our findings suggest that CMPK2 expression is associated with IRF3 activity, and that IRF3-dependent upregulation of CMPK2 may contribute to the formation of a positive feedback mechanism that amplifies mitochondrial stress, mtDNA release, and pro-inflammatory signaling. Importantly, genetic or pharmacological modulation of this IRF3-CMPK2-STING axis suppresses neuroinflammatory activation within the spinal cord and alleviates NP-like behaviors (Fig. 12). Together, these findings support a model in which CMPK2-associated mitochondrial immunometabolic dysregulation, operating prominently in microglia but within a broader spinal cord cellular network, contributes to sustained neuroinflammatory amplification in NP.

Although CMPK2 has previously been implicated in inflammatory conditions such as metabolic dysfunction-associated steatohepatitis (MASH) and intestinal inflammation [36, 37], its role in NP has remained largely unexplored. Here, bioinformatic analyses revealed robust upregulation of *Cmpk2* in SDH tissue from NP models as well as in LPS-stimulated BV2 microglia. Single-cell RNA sequencing and immunofluorescence co-staining in SNI mice demonstrated that CMPK2 is expressed in multiple spinal cord cell types, with relatively higher enrichment observed in microglia within the SDH. Consistently, LPS exposure markedly increased *Cmpk2* expression in BV2 or primary microglial cells in vitro. These observations are in agreement with a recent study by Yuan et al., which reported elevated *Cmpk2* expression in the chronic constriction injury model and confirmed its prominent association with microglial cells by immunofluorescence [38].

Mitochondria play a critical role in diverse cellular processes, encompassing energy metabolism, oxidative stress and inflammation [39]. Recent studies have suggested that CMPK2 not only facilitates mitochondrial DNA synthesis but also promotes the release of mtDNA into the cytosol [29, 31]. Yu et al. reported that mtDNA release mediated by the FOT-CMPK2 axis drives synovial inflammation and cartilage damage, while Li et al. showed that CMPK2-dependent mtDNA release contributes to house dust mite-induced allergic rhinitis. Extending these observations to the context of NP, our data show that AAV-mediated *Cmpk2* knockdown in SNI mice is associated with reduced cytosolic mitochondrial genome D-loop copy numbers in the SDH. Consistently, in LPS-stimulated BV2 microglia, silencing of *Cmpk2* markedly decreased both cytosolic double-stranded DNA fluorescence intensity and mtDNA D-loop copy numbers, indicating that CMPK2 is a critical regulator of mtDNA leakage in microglia under inflammatory conditions. In addition to mtROS generation and mtDNA leakage, mitochondrial membrane potential measurements in primary microglia further support a functional role of CMPK2 in regulating mitochondrial integrity during neuroinflammation.

As a prototypical mtDAMP, cytosolic mtDNA activates downstream innate immune signaling through engagement of the DNA sensor cGAS. In the SNI model, we observed upregulation of cGAS, STING, and p-IRF3 in SDH. Importantly, AAV-mediated knockdown of CMPK2 was associated with attenuation of this signaling response, supporting a role for cytosolic mtDNA release in linking CMPK2 activity to cGAS-STING-driven neuroinflammation. In vitro, LPS stimulation of BV2 and primary microglia similarly induced activation of the cGAS-STING-IRF3 axis, which was reduced by CMPK2 knockdown, further suggesting that CMPK2-dependent regulation of mitochondrial integrity and innate immune signaling is conserved across immortalized and primary microglial systems. Accumulating evidence supports the involvement of cGAS-STING signaling in multiple models of NP. In a rat model of chronic post-surgical pain induced by skin/muscle incision and retraction (SMIR), Yao et al. demonstrated activation of the cGAS-STING pathway, and intrathecal administration of the selective STING antagonist C-176 significantly reversed mechanical hypersensitivity [40]. Similarly, Mei et al. reported progressive upregulation of STING expression in the SNI mouse model, with pharmacological STING inhibition alleviating mechanical allodynia [41]. Emerging consensus suggests that chronic pain engages cGAS-STING signaling through diverse upstream triggers, including pathogen-derived DNA, self-DNA released from damaged cells, and cooperative activation of other pattern-recognition receptors such as Toll-like receptors [42]. Notably, recent studies also indicate a context-dependent, bidirectional role of cGAS-STING signaling in nociceptive processing, with both pro- and anti-nociceptive effects reported. Elucidating the molecular determinants underlying this functional duality remains an important direction for future investigation.

Having identified a role for CMPK2 in regulating mtDNA release and cGAS-STING-IRF3 activation in microglial cells, we next investigated whether upstream signaling events may reciprocally influence CMPK2 expression. Importantly, our analyses extend beyond the downstream consequences of CMPK2 activation and suggest that p-IRF3, a key effector of cGAS-STING signaling, may contribute to the regulation of *Cmpk2* expression. This IRF3-associated regulation of CMPK2 is consistent with a potential positive feedback mechanism, in which STING activation promotes IRF3 phosphorylation, IRF3 may enhance *Cmpk2* transcription, and elevated CMPK2 activity in turn may facilitate mtDNA release and sustained cGAS-STING signaling. Such a feedforward process may contribute to the persistence and amplification, rather than the initial triggering, of microglial neuroinflammation observed in NP. While previous studies in macrophages have suggested that IRF3 may regulate CMPK2 expression, our findings extend these observations by supporting the existence of a similar regulatory relationship in microglial cells under inflammatory conditions [32]. Notably, although our bioinformatic analyses prioritized IRF3 as a candidate regulator of CMPK2, other inflammatory transcription factors, such as IRF1 or NF-κB, may also participate in CMPK2 regulation depending on the pathological context. A systematic dissection of their relative contributions will be an important subject for future investigation.

Given the potential involvement of the IRF3-CMPK2-STING-associated feedback mechanism in sustaining neuroinflammatory signaling in NP, our findings suggest that CMPK2 may represent a potential therapeutic target. Through molecular docking and molecular dynamics simulations, we evaluated the interaction between CMPK2 and NDGA, which showed a high predicted binding affinity. NDGA, a major bioactive metabolite derived from the chaparral shrub, has been reported to exert biological effects in diverse pathological contexts, including neurological disorders, cancer, and viral infections [43–45]. Notably, prior studies showed that NDGA attenuates carrageenan-induced thermal and mechanical hyperalgesia, although the underlying molecular mechanism remained unclear [46]. In the present study, post-injury administration of NDGA in SNI mice was associated with reduced expression of key components of the IRF3-CMPK2-STING axis, including cGAS, STING, and p-IRF3, decreased cytosolic mtDNA release, attenuated pro-inflammatory cytokine production, and improvement in mechanical allodynia. These pharmacological effects showed a similar trend to those observed following genetic Cmpk2 knockdown, supporting a role for CMPK2-associated signaling in mediating these responses.

Although NDGA is a polyphenolic small molecule with well-documented antioxidant and anti-inflammatory activities, accumulating evidence suggests CMPK2 as a direct molecular target of NDGA. In an ischemic stroke model, NDGA was shown to inhibit CMPK2 kinase activity in a concentration-dependent manner, and surface plasmon resonance (SPR) analysis demonstrated direct binding between NDGA and CMPK2 [43]. Similarly, a recent study of MASH identified NDGA as the most potent CMPK2 inhibitor from a high-throughput compound library and further confirmed high-affinity NDGA-CMPK2 binding by SPR [36]. Consistent with these biochemical findings, NDGA treatment in our study phenocopied genetic CMPK2 suppression, including reduced mtDNA release, inhibition of cGAS-STING-IRF3 signaling, and attenuation of NP-like behaviors. Collectively, these concordant pharmacological and genetic observations support the involvement of CMPK2-associated signaling in mediating the effects of NDGA in the context of NP. Nevertheless, given the pleiotropic nature of NDGA, future studies employing genetic rescue strategies or more selective CMPK2 inhibitors will be required to further define target specificity in vivo.

In this study, genetic *Cmpk2* knockdown was employed primarily for mechanistic validation, enabling efficient suppression of CMPK2 to assess its contribution to NP-related processes. By contrast, potential therapeutic relevance was evaluated using post-injury pharmacological inhibition, which demonstrated that targeting CMPK2-associated signaling is associated with improvement in established NP-like behaviors. Together, these complementary genetic and pharmacological approaches provide convergent evidence supporting both the mechanistic involvement and potential translational relevance of CMPK2 in NP. Additionally, intrathecal administration of NDGA was used as a proof-of-concept strategy to interrogate spinal neuroimmune mechanisms and evaluate therapeutic effects in vivo, rather than to model systemic clinical dosing.

Despite the strengths of this study, several limitations should be acknowledged. First, NP is a complex, multicomponent pathological process involving coordinated changes in peripheral nerves, dorsal root ganglia (DRG), immune cell trafficking, and central sensitization. In the present study, we primarily focused on the spinal dorsal horn as a key integrative hub for neuroimmune signaling and pain processing. Although peripheral nerve and DRG compartments were not directly examined, our findings identify a central immunometabolic amplification mechanism within the spinal cord, with prominent microglial involvement, that may contribute to sustained neuroinflammation and central sensitization. Future studies incorporating parallel analyses of peripheral and central compartments will be required to more fully elucidate mechanisms of peripheral-central coupling in NP. Second, although BV2 microglial cells provide a widely used and practical in vitro model, they may not fully recapitulate the phenotypic and functional heterogeneity of primary microglia in vivo. Therefore, mechanistic conclusions derived from BV2 cells should be interpreted with caution. While we incorporated primary microglia experiments to enhance physiological relevance, future studies employing microglia-specific genetic models will be important to further strengthen cell-type–resolved conclusions. In addition, intrathecal AAV-mediated gene silencing does not confer strict cell-type specificity and may affect multiple neural and glial populations. Accordingly, the present in vivo genetic approach was intended for mechanistic target validation rather than definitive microglia-specific causal inference. Third, although the core components of the mtDNA-cGAS-STING-IRF3-CMPK2 axis are evolutionarily conserved, validation of this pathway in human NP tissues will be essential to establish clinical relevance. Moreover, NDGA treatment was associated with reduced CMPK2-related signaling and attenuation of NP-like behaviors. However, as a pleiotropic natural compound, off-target effects cannot be excluded. The development of more selective CMPK2 inhibitors, together with complementary genetic approaches, will be required to further delineate the specific contribution of CMPK2 to the observed in vivo effects. Finally, the upstream mechanisms governing mitochondrial reactive oxygen species-mediated mtDNA oxidation and its subsequent cytosolic release were not directly addressed in this study. Further investigation of these processes may provide deeper insight into the molecular events linking mitochondrial dysfunction to innate immune activation in NP.

## Conclusions

This study supports the existence of an IRF3-CMPK2-STING-associated immunometabolic feedback mechanism that may be involved in linking mitochondrial metabolic dysregulation to sustained innate immune signaling within the spinal cord microenvironment during NP, with microglia representing a major responsive cellular population. By providing insight into how mitochondrial stress may promote persistent mtDNA-driven inflammatory signaling, our findings contribute to a better understanding of the chronicity of neuroinflammation and pain. Importantly, these results identify mitochondrial immunometabolism as a potential therapeutic axis and suggest that modulation of CMPK2-associated signaling pathways may represent a potential strategy for the treatment of NP and other neuroinflammatory conditions characterized by prolonged innate immune activation.

## Supplementary Information

Below is the link to the electronic supplementary material.

Supplementary Material 1

Supplementary Material 2

Supplementary Material 3

Supplementary Material 4

## Data Availability

The data supporting this research are available upon reasonable request from the corresponding author.

## Abbreviations

NP: Neuropathic pain
SDH: Spinal dorsal horn
ROS: Reactive oxygen species
mtDNA: Mitochondrial DNA
mtDAMP: Mitochondrial danger-associated molecular pattern
cGAS: Cyclic GMP-AMP synthase
STING: Stimulator of interferon genes
TBK1: TANK-binding kinase 1
IRF3: Interferon Regulatory Factor 3
CMPK2: Cytosine/uridine monophosphate kinase 2
PAMPs: Pathogen-associated molecular patterns
dsDNA: Double-stranded DNA
dsRNA: Double-stranded RNA
LPS: Lipopolysaccharide
DEGs: Differentially expressed genes
AAV: Adeno-associated virus
ChIP: Chromatin immunoprecipitation
GEO: Gene Expression Omnibus
UMAP: Uniform Manifold Approximation and Projection
SNI: Spared nerve injury
PWT: Paw withdrawal threshold
NDGA: Nordihydroguaiaretic acid
qRT-PCR: Quantitative real-time PCR
MD: Molecular dynamics
RMSD: Root-mean-square deviation
RMSF: Root-mean-square fluctuation
FEL: Free-energy landscape
SASA: Solvent-accessible surface area
