# Supplementary material for: Mitochondrial DNA release contributes to neuropathic pain via a cGAS-STING-IRF3-CMPK2-associated immunometabolic feedback mechanism
Source: J Transl Med. 2026 May 22;24:920. doi: 10.1186/s12967-026-08314-8 (PMC13374242; doi:10.1186/s12967-026-08314-8)
Supplement: Supplementary file 1 — Supplementary Material 1 [file 12967_2026_8314_MOESM1_ESM.docx]

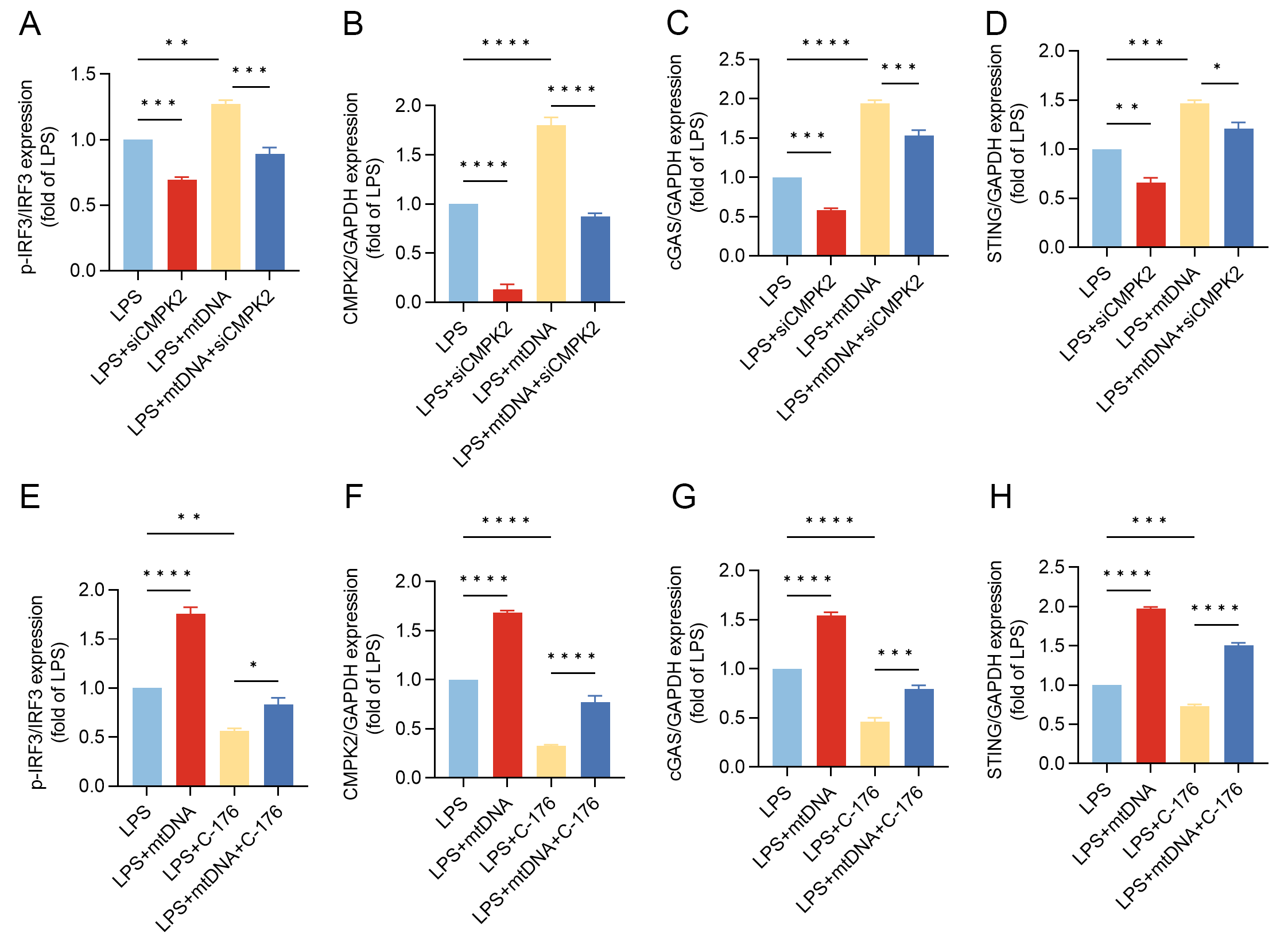


**Fig. S1** Quantitative analysis of CMPK2, cGAS, STING, and p-IRF3 protein expression in mtDNA rescue and C-176 inhibition experiments of Fig. 7.
