# Supplementary material for: Mitochondrial DNA release contributes to neuropathic pain via a cGAS-STING-IRF3-CMPK2-associated immunometabolic feedback mechanism
Source: J Transl Med. 2026 May 22;24:920. doi: 10.1186/s12967-026-08314-8 (PMC13374242; doi:10.1186/s12967-026-08314-8)
Supplement: Supplementary file 2 — Supplementary Material 2 [file 12967_2026_8314_MOESM2_ESM.docx]

**Supplementary Table 1. Primer sequences used in qRT-PCR and target genes knockdown.**

| Primers | Sequence (forward/reverse, 5'-3') | Usage |
| --- | --- | --- |
| mtDNA Dloop1 | AATCTACCATCCTCCGTGAAACC  TCAGTTTAGCTACCCCCAAGTTTAA | qPCR |
| mtDNA Dloop2 | CCCTTCCCCATTTGGTCT  TGGTTTCACGGAGGATGG | qPCR |
| mtDNA Dloop3 | TCCTCCGTGAAACCAACAA  AGCGAGAAGAGGGGCATT | qPCR |
| nDNA Tert | CTAGCTCATGTGTCAAGACCCTCTT  GCCAGCACGTTTCTCTCGTT | qPCR |
| IL-1β | TTGACGGACCCCAAAAGATG  CAGCTTCTCCACAGCCACAA | qPCR |
| TNF-α | CCAAATGGCCTCCCTCTCAT  TGGTGGTTTGCTACGACGTG | qPCR |
| IL-6 | CTGCAAGAGACTTCCATCCAG  AGTGGTATAGACAGGTCTGTTGG | qPCR |
| CCL5 | GCTGCTTTGCCTACCTCTCC  TCGAGTGACAAACACGACTGC | qPCR |
| CFB | CAAGCAGCACAAGGAACAGT  CCTTGGGCCTTTGTAGCATC | qPCR |
| GBP2 | GGGGTCACTGTCTGACCACT  GGGAAACCTGGGATGAGATT | qPCR |
| IL1b | TGCCACCTTTTGACAGTGATG  AAGGTCCACGGGAAAGACAC | qPCR |
| RSAD2 | AAGCGTGGCGGAAAGTA  GGCGTGAATGTCCCTGT | qPCR |
| CMPK2 | GGCAATTATCTCGT GGCTTC  GTAGCTATGGCGTAGGTGGC | qPCR |
| IFIT1 | TGAACAACAAGACCCTCGTG  TTCAGTTTGTAGACTAGCCCAAG | qPCR |
| GAPDH | TGTGTCCGTCGTGGATCTGA  CCTGCTTCACCACCTTCTTGA | qPCR |
| si-CMPK2#1 | CAACUUUCCUGUUAUUGUATT  UACAAUAACAGGAAAGUUGTT | siRNA |
| si-CMPK2#2 | GGAAGAUCUUUGAUGAUGATT  UCAUCAUCAAAGAUCUUCCTT | siRNA |
| si-CMPK2#3 | GGCAGUACUUGACCUAGUUTT  AACUAGGUCAAGUACUGCCTT | siRNA |
