# Supplementary material for: Mitochondrial DNA release contributes to neuropathic pain via a cGAS-STING-IRF3-CMPK2-associated immunometabolic feedback mechanism
Source: J Transl Med. 2026 May 22;24:920. doi: 10.1186/s12967-026-08314-8 (PMC13374242; doi:10.1186/s12967-026-08314-8)
Supplement: Supplementary file 3 — Supplementary Material 3 [file 12967_2026_8314_MOESM3_ESM.docx]

Supplementary Table 2. Antibodies used in WB, IF and CHIP.

| **Antibody** | **Origin** | **Catalog #** | **Assay** |
| --- | --- | --- | --- |
| CMPK2 | OriGene | TA320098 | 1:100 IF  1:1000 WB |
| IRF3 | Abcam | ab76409 | 1:1000 WB  5µg CHIP |
| p-IRF3 | Proteintech | 29528-1-AP | 1:1000 WB |
| Sting | Cell Signaling Technology | 13647 | 1:1000 WB |
| cGAS | Cell Signaling Technology | 31659 | 1:1000 WB |
| GAPDH | Beyotime | AF0006 | 1:1000 WB |
| IBA-1 | Wako | 011-27991 | 1:100 IF |
| GFAP | CST | #3670 | 1:100 IF |
| NeuN | Abcam | ab104224 | 1:100 IF |
