# Supplementary material for: Mitochondrial DNA release contributes to neuropathic pain via a cGAS-STING-IRF3-CMPK2-associated immunometabolic feedback mechanism
Source: J Transl Med. 2026 May 22;24:920. doi: 10.1186/s12967-026-08314-8 (PMC13374242; doi:10.1186/s12967-026-08314-8)
Supplement: Supplementary file 4 — Supplementary Material 4 [file 12967_2026_8314_MOESM4_ESM.docx]

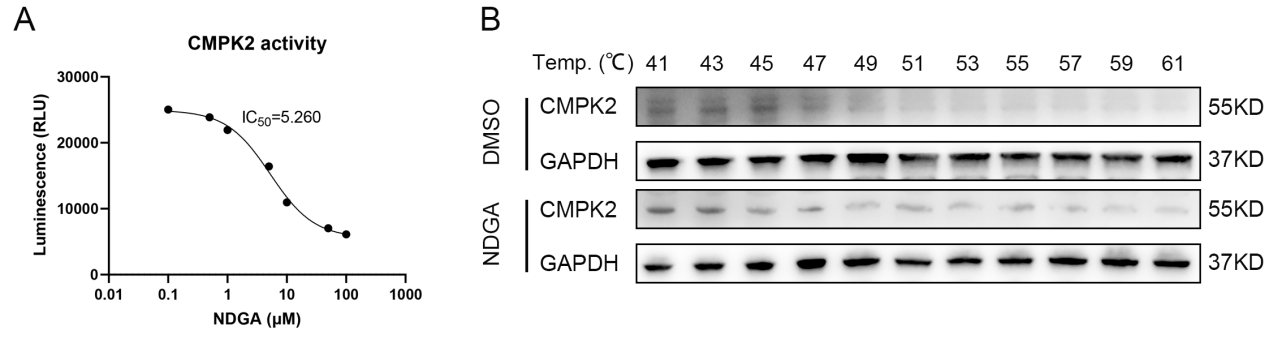


**Fig. S2** NDGA inhibits CMPK2 enzymatic activity and increases the thermal stability of CMPK2. (A) In vitro CMPK2 kinase activity assay showing concentration-dependent inhibition of CMPK2 activity by NDGA. The calculated IC₅₀ value was 5.26 μM. (B) Cellular thermal shift assay (CETSA) analysis of CMPK2 in BV2 cells treated with DMSO or NDGA (10 μM). Cell lysates were heated at the indicated temperatures, and soluble CMPK2 protein levels were analyzed by Western blot. GAPDH was used as a loading control.
